# Greenness and chronic respiratory health issues: a systematic review and meta-analysis

**DOI:** 10.3389/fpubh.2023.1279322

**Published:** 2023-12-06

**Authors:** Mingcheng Tang, Wei Liu, Haifang Li, Fengyi Li

**Affiliations:** ^1^School of Landscape Architecture and Forestry, Qingdao Agricultural University, Qingdao, China; ^2^School of Art, Qufu Normal University, Rizhao, Shandong, China

**Keywords:** greenness, chronic respiratory disease, normalized difference vegetation index, meta-analysis, systematic review

## Abstract

**Introduction:**

The number of chronic respiratory disease (CRD) individuals worldwide has been continuously increasing. Numerous studies have shown that greenness can improve chronic respiratory health issues through different mechanisms, with inconsistent evidence. By quantitatively summarizing existing studies, our purpose is to determine the connection between greenness exposure and various chronic respiratory health.

**Methods:**

We conducted a comprehensive search on PubMed, EMBASE, and Web of Science core databases to identify relevant studies on the correlation between greenness exposure and chronic respiratory health issues. Studies published up to January 2023 were included in the search. The study used the most frequent indicator (normalized difference vegetation index [NDVI]) as the definition of greenness exposure.

**Results:**

We finally identified 35 studies for meta-analysis. We calculated pooled effects across studies using a random-effects model and conducted a subgroup analysis by age and buffer zones to discuss the effects on chronic respiratory health issues. This study showed that 0.1 increments in NDVI were significantly related to lower rates of asthma incidence, lung cancer incidence, and chronic obstructive pulmonary disease (COPD) mortality risk; the pooled RRs were 0.92 (95% CI: 0.85–0.98), 0.62 (95% CI: 0.40–0.95), and 0.95 (95% CI: 0.92– 0.99), respectively. For the age subgroup, the higher greenness exposure level was related to the incidence rate of asthma among teenagers aged 13–18years (RR: 0.91; 95% CI: 0.83–0.99). For the buffer subgroup, a positive relationship with greenness exposure and asthma incidence/prevalence at 200–300m and 800– 1000m buffers, as well as the COPD mortality at 800–1000m buffer, the pooled RRs were 0.92 (95% CI: 0.86–0.98), 0.87 (95% CI: 0.81–0.93), and 0.93 (95% CI: 0.88– 0.98), respectively. Evidence of publication bias was not detected in this study.

**Discussion:**

Our study is the first global meta-analysis between greenness and various CRDs to report an inverse association. Further research is needed in order to determine the effect of greenness exposure on different CRDs. Therefore, when planning for green development, more consideration must be given to public health and green management as intervention measures.

https://www.crd.york.ac.uk/PROSPEROFILES/384029_STRATEGY_20230116.pdf

## 1 Introduction

Chronic respiratory diseases (CRDs) have already become a non-negligible cause of death globally. The disability-adjusted life years (DALYs) counts of CRDs significantly increased from 1990 to 2017. Over the past 30 years, 544.9 million individuals worldwide have been diagnosed with CRDs (1). Among the most common CRDs are allergic rhinitis (AR), chronic obstructive pulmonary disease (COPD), lung cancer, asthma, non-cystic fibrosis bronchiectasis (NCFBE), cystic fibrosis (*CF*), and idiopathic pulmonary fibrosis (IPF) (2–4), which were the main causes of CRD-related incidence and mortality worldwide (5). CRD incidence is growing globally, posing a serious threat to human health and causing heavy financial burdens (6).

CRDs are caused by a variety of factors, including indoor and outdoor environmental exposure, interplays between genetic predispositions, and sociodemographic features throughout the lifespan (7). A series of environmental risk factors related to chronic respiratory diseases have been investigated, including outdoor air pollutants (8), urban heat islands (UHI) (9, 10), biodiversity loss (11), noise pollution (12, 13) indoor mold and humidity (14), tobacco smoke (15), renovation activities (16) and household pets (17). As urbanization continues to increase worldwide, more and more people are exposed to urban-related environmental hazards (18). Several studies have suggested that greenness can benefit chronic respiratory health issues through the purification of air, the alleviation of the heat island effect, the increase of microbial diversity, and the encouragement of physical activity (19–24).

A large number of studies have confirmed that greenness has positive effects on the prevention and treatment of CRD incidence and mortality, including asthma (25–27), AR (28), COPD (29, 30), and lung cancer (31, 32). A number of reviews and meta-analyses have been conducted to summarize the results of existing studies. Seven meta-analyses explored the correlation between greenness and chronic respiratory health outcomes, including asthma (33–36), AR (33–37), respiratory symptoms, diseases, allergies (38), and cancer incidence/prevalence/mortality (39). The relationship of greenness with asthma and AR was the most frequently investigated. However, the findings of the abovementioned reviews are inconsistent. Qiu et al. (34) reported that asthma and AR were significantly prevented by high-level residential greenness, while Parmes et al. (38) found that the odds of asthma and AR increased significantly by 5.9–13.0% for every 10% increase in greenness coverage. It has been recognized in recent years that research evidence regarding the influence of greenness exposure on health outcomes in CRDs has been growing; however, systematic reviews and meta-analyses concentrated only on one or two particular health outcomes in CRDs. Furthermore, no generalized systematic reviews and meta-analyses have been conducted to summarize studies on various CRDs, and no precise and global estimate has been made on the reduction in CRD incidence/prevalence and mortality rate correlated with greenness exposure. Therefore, it is necessary to conduct comprehensive research involving more CRD outcomes.

Millions of people of all ages suffer from CRDs, which are regarded as major causes of morbidity, death, economic burden, and social problems all over the world (40). Taking asthma as an example, asthma prevalence peaks between the ages of 5 and 9 (1) Some patients develop asthma when they are adults for the first time (adult-onset asthma) (41). Evidence of the connection between greenness exposure and CRDs has grown over the past decade, but systematic reviews and meta-analyses have focused on particular age groups, such as children and adolescents (33, 36–38). However, there is a lack of horizontal comparisons between different age groups in regard to greenness exposure and CRDs.

Buffer zones of different radii were used to evaluate greenness exposure. The most commonly used indicator is the normalized difference vegetation index (NDVI) (42). However, different buffer zones of NDVI suggest different ways in which greenness affects health (10). NDVI within 500 m of a residential address can be used to indicate the immediate neighborhood greenness of a residence appropriate for physical activity (43, 44), decreasing noise and air pollution (45, 46). NDVI with smaller buffers (e.g., 200–300 m) indicates the visual impact of greenness seen from home (27). NDVI with a larger buffer (e.g., 800–1,000 m) may suggest that the area is suitable for recreational purposes and can be visited by people (47). Some studies have explored correlations between greenness exposure in different buffer zones and CRD risk (48–54). In larger buffers, Zeng et al. (54) observed stronger associations between asthma and school greenness, while Dadvand et al. (50) suggested higher relative prevalences of current allergic rhinoconjunctivitis and asthma for surrounding greenness. In addition, Xiao et al. (53) reported stronger correlations with the odds of COPD for NDVI in a 1,000 m buffer. Bereziartua et al. (49) found that increments of NDVI inside both a 300 m grid and a 1,000 m buffer were not associated with respiratory diseases. Fan et al. (55) discovered that neighborhood greenness had a significant positive correlation with COPD prevalence across different NDVI buffer sizes. Up to now, three systematic reviews and meta-analyses have carried out grouping analyses in different buffers of asthma and AR (34, 35, 37) A greater impact of residential greenness has been observed on the odds of respiratory system diseases in the larger buffer zones (e.g., asthma and AR) (34). Health outcomes for various CRDs are unknown as a result of different greenness buffer zones.

Therefore, we systematically conducted a meta-analysis of published studies to examine the correlation between greenness exposure and a broad range of chronic respiratory health issues. We also investigated the health impacts of different age groups and different greenness buffers, providing healthcare professionals and researchers with a more generalized and higher-quality piece of evidence on the connection between greenness exposure and chronic respiratory health issues.

## 2 Materials and methods

### 2.1 Search strategy and data sources

We followed standard protocols suggested by the PRISMA (Preferred Reporting Items for Systematic Reviews and Meta-Analyses) guidelines for the study (Supplementary Table S1) (56). In addition, our study protocol has been registered at PROSPERO (registered number: CRD42023384029). For the study, the Embase, Web of Science, and PubMed databases were searched to find epidemiological studies about greenness exposure and chronic respiratory health issues. The English-language search was conducted with the last update on 31 January 2023. We used combinations of terms concerning greenness (e.g., greenness, green space, and NDVI) and CRD incidence/prevalence/mortality (e.g., pulmonary phthisis, asthma, chronic obstructive pulmonary disease, and rhinitis). To find more potentially pertinent studies, we also conducted manual searches for reference lists of all pertinent systematic reviews and eligible studies. The detailed search strategy and pertinent search outcomes are shown in Supplementary Table S2.

### 2.2 Study selection and eligibility criteria

Six criteria were required for studies to be eligible: (i) conducted among the general population; (ii) examined the connections between greenness and CRD incidence/prevalence or mortality; (iii) were cross-sectional, case–control, cohort, and ecological study designs; (iv) assessed greenness exposure with objective metrics based on continuous NDVI rather than classification based on measurement data (e.g., quartiles and tertiles). We chose an exposure index NDVI for comparisons between studies since it is an excellent target of overall greenness and is the most commonly used definition in the selection of studies; (v) evaluated the health outcomes of CRDs based on a clinical evaluation (e.g., International Classification of Primary Care[ICPC], International Classification of Diseases [ICD], self-reported physis diagnoses history, death records, and medical records related to respiratory situations); and (vi) written in English. We excluded reports that included greenness as a confounding factor or that lacked essential details. The references identified in three databases were imported into Endnote 20. The remaining articles were independently assessed through the title and abstract by two researchers (M.T. and F.L.) after the duplicates were removed, and disagreements were resolved by H.L. and W.L.

### 2.3 Data extraction

The following data from eligible studies were extracted independently by two reviewers (M.T. and F.L.). Data extracted from each study included: year of publication, lead author, location (country/region), type of study, number and age range of samples, NDVI buffer, exposure unit reported, respiratory health issues assessed, results sourced, estimates of risk with a 95% CI, and confounders. During the review process, a third reviewer (H.L.) was consulted to address any inconsistencies in data extraction.

### 2.4 Assessment of the risk of bias

The RoB of the included studies was assessed by two independent investigators (M.T. and W.L.), and any disagreement was addressed through discussion with two other investigators (F.L. and H.L.). In order to estimate the RoB of selected studies, we consulted a checklist developed by WHO (57) and van Kempen [(58), Tables S4, 5]. Based on the tool, we evaluated the RoB associated with exposure assessments, participants’ selection, confounding, and health outcomes.

### 2.5 Scientific evidence levels

The Grading of Recommendations, Assessments, Developments, and Evaluations (GRADE) standard was used to grade the credibility of the pooled evidence. Subsequently, each combination of exposure outcomes was assessed for five factors (59). We used GRADE Profiler 3.6 to grade the confidence rating into four descriptors: “high,” “moderate,” “low,” or “very low.” The GRADE method for evaluating the quality of evidence refers to the report by Welsh et al. (60).

### 2.6 Data standardization and meta-analysis methods

This study mainly focused on the connection between greenness exposure and CRD incidence and mortality, as well as the subgroup analysis of age groups and buffer groups. For the overall meta-analysis and age subgroup analysis, we selected the pooled effect obtained from the largest buffer (within 1,000 m buffer) in the study and estimated the associations between CRD outcomes with different age groups and greenness exposure in adults aged 0–7 years, 8–12 years, 13–18 years, 19–40 years, and over 40 years independently based on the classification of all age groups included in the study. Moreover, we estimated the associations between CRD outcomes and NDVI at 200–300 m, 400–500 m, and 800–1,000 m buffers. A meta-analysis of chronic respiratory health risk associations with greenness was conducted using all odds ratios (ORs), risk ratios (RRs), and hazard ratios (HRs). The outcomes of interest (OR, RR, and HR) were included in the same meta-analysis in accordance with previous practice (61). This is acceptable in this situation because the outcome of interest is relatively common, but its effect size is small (62). In this study, we evaluated the effects of exposure to greenness on CRD outcomes by calculating the pooled RR (63). In order to be able to compare between studies, the size of the effects used in the meta-analysis was normalized and scaled to the same size (NDVI increased by 0.1). Pooled effect estimates were calculated as follows:


$$OR_{\left(s\right)}=O{R_{\left(o\right)}}^{\mathrm{incremen}t_{\left(s\right)}/\mathrm{incremen}t_{\left(o\right)}}$$

(substituting RR or HR as applicable).

where *OR_(s)_* represented the standardized effect estimates, *OR_(o)_* represented the original effect estimates, *increment_(s)_* represented the standardized increases of NDVI (per 0.1-unit increase), and *increment_(o)_* represented the original increases in NDVI (42).

For this study, the “Review Manager 5.4” software was used, and a *p*-value <0.05 was taken as statistically significant. All results were presented as forest plots with 95% confidence intervals. In order to evaluate the impact of individual studies, every study was excluded one at a time during the sensitivity analysis. It was decided to use the random effect model in this study because it was a conservative method for high heterogeneity research (64). *I*^2^ statistics were used to evaluate the heterogeneity between studies (*I*^2^ = 0–25% signified no heterogeneity; *I*^2^ = 25–50% signified low heterogeneity; *I*^2^ = 50–75% signified moderate heterogeneity; *I*^2^ = 75–100% signified high heterogeneity) (65).

## 3 Results

### 3.1 Retrieval of literature and characteristics of the study

**Figure 1:**
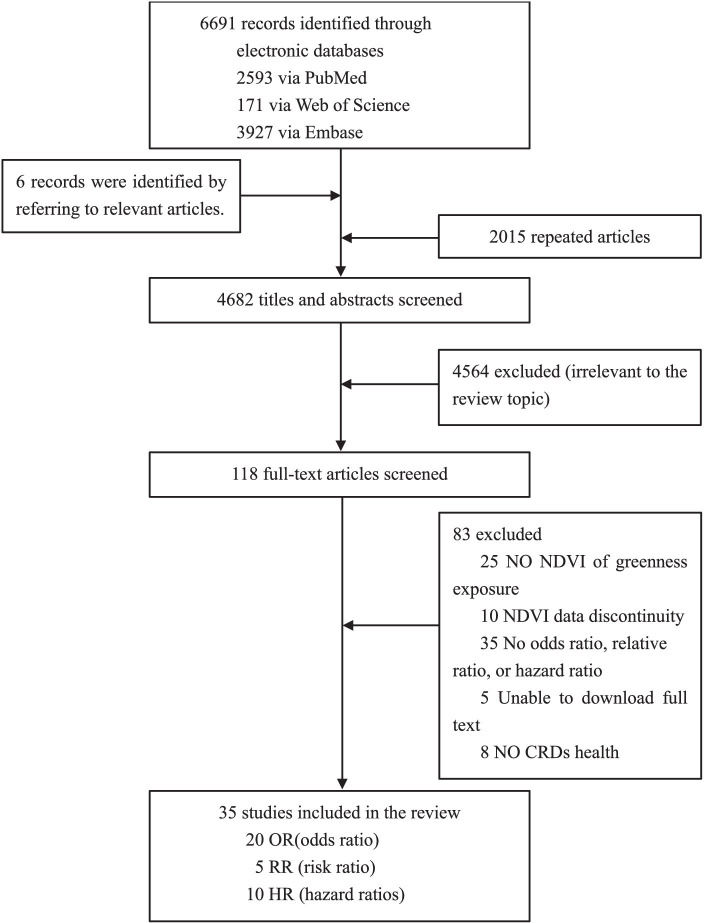
Flowchart of literature selection.

As shown in Figure 1, according to our search strategy, a total of 6,691 relevant studies were retrieved from three databases, and another six studies were manually added to the reference list of review studies. We assessed the titles and abstracts of 4,682 studies after removing duplicates. Next, the full texts of 118 studies were retrieved, and we further excluded 83 studies that were irrelevant. Finally, we selected 35 studies for meta-analysis, of which 20 had a pooled effect value of OR, 5 had a pooled effect value of RR, and 10 had a pooled effect value of HR.

In Supplementary Table S3, the 35 studies were summarized according to their characteristics. The studies were all published in the last 10 years, indicating a growing interest in chronic respiratory health issues and greenness. The sample populations of this study were drawn from 20 countries, of which 15 (43%) were in Europe, 15 (43%) in Asia, and 5 (14%) in North America. There were 10 cross-sectional studies, 18 cohort studies, 4 ecological studies, and 3 case–control studies. There was a wide variation in the statistical sample size of these studies, from 478 children (66) to 10,481,566 aged over 30 years (31).

### 3.2 Primary outcomes

#### 3.2.1 NDVI and CRD incidence or prevalence

A total of 27 studies analyzed the connection between greenness exposure and CRD incidence/prevalence, including 14 cohort studies (1, 25, 26, 32, 52, 66–74), 10 cross-sectional studies (30, 45, 51, 53–55, 75–78), and 3 case–control studies (48, 79, 80).

Among these studies, 12 studies (44%) found that increasing NDVI and CRD incidence/prevalence in the overall samples had a significant beneficial effect, while 2 studies (7%) showed that an increment in NDVI was related to a slight increase in CRD incidence/prevalence. Eight studies found a stronger association between younger individuals (26, 54, 55), low urbanicity areas (25, 30, 81), low humidity and high-temperature areas (71), and lower household income participants (25, 82). There were no statistically significant connections found in the remaining 13 studies (49%).

**Figure 2:**
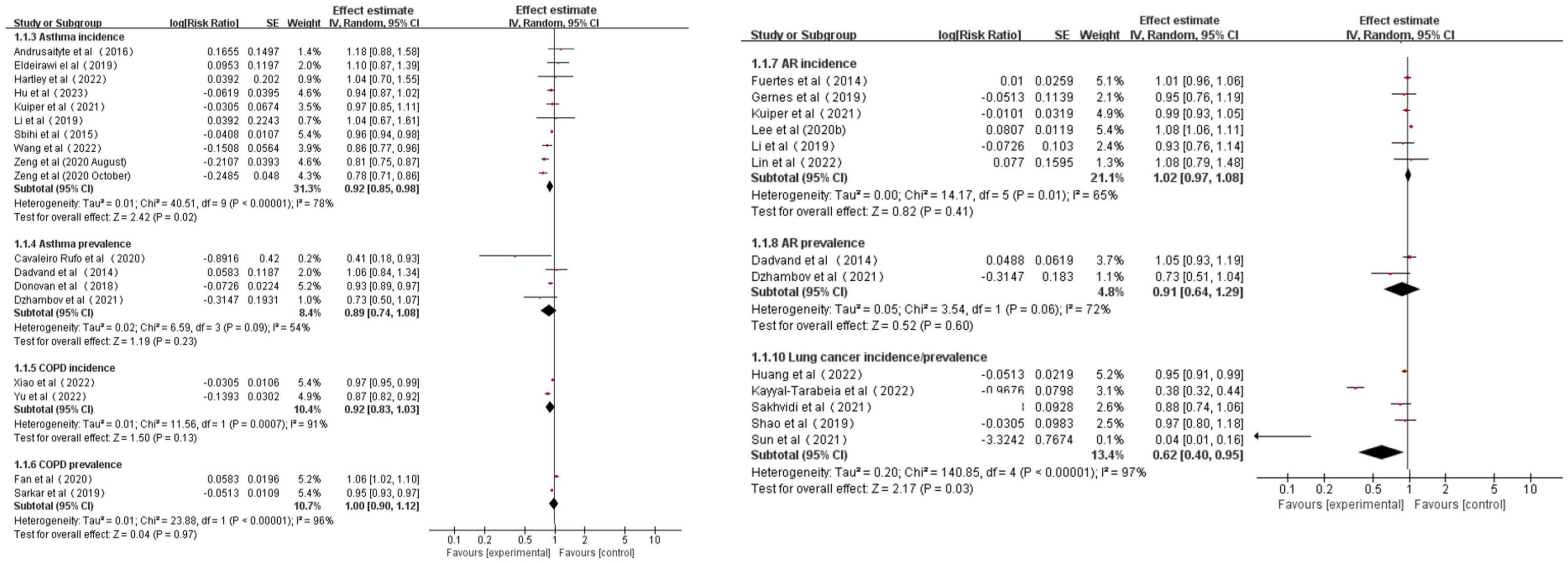
Meta-analysis of CRD incidence/prevalence for 0.1 increments of NDVI.

According to the pooled results, for every 0.1 increase in NDVI, the pooled RRs for asthma, AR, COPD, and lung cancer incidence were 0.92 (95% CI: 0.85–0.98, *p* = 0.02, *I*^2^ = 78%), 1.02 (95% CI: 0.97–1.08, *p* = 0.41, *I*^2^ = 65%), 0.92 (95% CI: 0.83–1.03, *p* = 0.13, *I*^2^ = 91%), and 0.62 (95% CI: 0.40–0.95, *p* = 0.03, *I*^2^ = 97%), respectively (Table 1 and Figure 2). The pooled RRs for asthma, AR, and COPD prevalence were 0.89 (95% CI: 0.74–1.08, *p* = 0.23, *I*^2^ = 54%), 0.91 (95% CI: 0.64–1.29, *p* = 0.60, *I*^2^ = 72%), and 1.00 (95% CI: 0.90–1.12, *p* = 0.97, *I*^2^ = 96%), respectively (Table 1 and Figure 2). High heterogeneity was observed for asthma incidence, COPD incidence/prevalence, and lung cancer incidence. Moderate heterogeneity was observed for asthma prevalence and AR incidence/prevalence. According to the GRADE system, asthma incidence and lung cancer incidence had “low” confidence, while the other CRDs had “very low” confidence in the pooled evidence (Supplementary Table S6).

**Table 1:** Summary of meta-analyses of studies concerning NDVI and CRD incidence /prevalence.

Outcomes	Studies (*n*)	Continuous exposure		GRADE
		Heterogeneity *I*^2^ (*p*-value)	Summary effect size (RR) (95% CI)	
Asthma incidence	9	78% (0.02)	**0.92 (0.85, 0.98)**	LOW
Asthma prevalence	4	54% (0.23)	0.89 (0.74, 1.08)	VERY LOW
AR incidence	6	65% (0.41)	1.02 (0.97, 1.08)	VERY LOW
AR prevalence	2	72% (0.60)	0.91 (0.64, 1.29)	VERY LOW
COPD incidence	2	91% (0.13)	0.92 (0.83, 1.03)	VERY LOW
COPD prevalence	2	96% (0.97)	1.00 (0.90, 1.12)	VERY LOW
Lung cancer incidence	5	97% (0.03)	**0.62 (0.40, 0.95)**	LOW

Weights are from random-effects analysis. When the 95% CI excludes 1 or 0, summary effect estimates are in bold. CRD, chronic respiratory disease; RR, relative risk; *I*^2^, heterogeneity statistic; CI, confidence interval; NDVI, normalized difference vegetation index; AR, allergic rhinitis; COPD, chronic obstructive pulmonary disease.

As a result of sensitivity analyses, we observed that most of the associations remained unchanged after any single study was excluded, which indicated that our findings were robust. However, the pooled estimates for COPD and lung cancer incidence became marginally significant after the exclusion of one study (Supplementary Table S9).

#### 3.2.2 NDVI and CRD mortality

Eight studies analyzed the connection between greenness exposure and CRD mortality, consisting of five cohort studies (31, 49, 83–85) and three ecological studies (86–88).

Three studies (38%) reported a significantly beneficial relationship between NDVI and CRD mortality. Two studies suggested that protective relationships appeared to be significant among women (85) and younger individuals (<65 years) (31, 85). No statistically significant association was reported in the remaining five (62%) studies.

**Figure 3:**
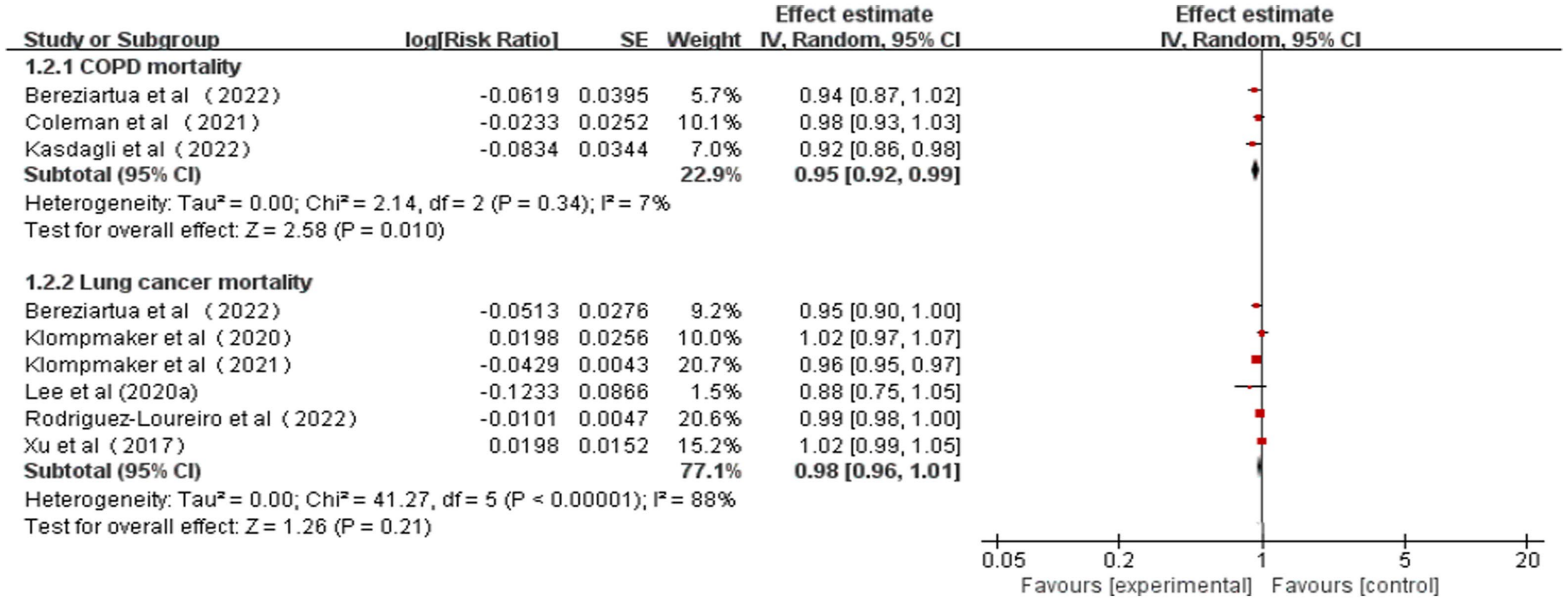
Meta-analysis of the relationship between greenness exposure and CRD mortality for 0.1 increments of NDVI.

The pooled RRs for COPD and lung cancer mortality (0.1 increments in NDVI) were 0.95 (95% CI: 0.92–0.99, *p* = 0.01, *I*^2^ = 7%) and 0.98 (95% CI: 0.96–1.01, *p* = 0.21, *I*^2^ = 88%), respectively (Table 2 and Figure 3). High heterogeneity emerged for lung cancer mortality, and no heterogeneity emerged for COPD mortality. According to the GRADE system, COPD mortality had “moderate” confidence and lung cancer mortality had “very low” confidence in the pooled evidence (Supplementary Table S6).

**Table 2:** Summary of meta-analyses of studies concerning NDVI and CRD mortality.

Outcomes	Studies (*n*)	Continuous exposure		GRADE
		Heterogeneity *I*^2^ (*p*-value)	Summary effect size (RR) (95% CI)	
COPD mortality	3	7% (0.01)	**0.95 (0.92, 0.99)**	MODERATE
Lung cancer mortality	6	88% (0.21)	0.98 (0.96, 1.01)	VERY LOW

Weights are from random-effects analysis. When the 95% CI excludes 1 or 0, summary effect estimates are in bold. CRD, chronic respiratory disease; RR, relative risk; *I*^2^, heterogeneity statistic; CI, confidence interval; NDVI, normalized difference vegetation index; COPD, chronic obstructive pulmonary disease.

As a result of sensitivity analyses, we observed that most of the associations remained unchanged after any single study was excluded, which indicated that our findings were stable. However, COPD mortality estimates became marginally significant after excluding one study owing to the limited number of included studies (Supplementary Table S10).

### 3.3 Subgroup analysis

#### 3.3.1 Age subgroups

Considering the age characteristics of the subjects in each study, meta-analyses were performed in five age subgroups (aged 0–7 years, 8–12 years, 13–18 years, 19–40 years, and over 40 years) (Table 3). Sufficient data on asthma, AR, and COPD incidence/prevalence was available to perform age subgroup analyses.

**Table 3:** Meta-analysis of age subgroups.

Outcomes	Studies (*n*)	Continuous exposure		GRADE
		Heterogeneity *I*^2^ (*p*-value)	Summary effect size (RR) (95% CI)	
**Aged 0–7 years**				
Asthma incidence/prevalence	4	51% (0.83)	0.98 (0.80, 1.20)	VERY LOW
AR incidence	2	0% (0.93)	0.99 (0.83, 1.19)	LOW
**Aged 8–12 years**				
Asthma prevalence	2	63% (0.60)	0.91 (0.63, 1.30)	VERY LOW
AR prevalence	2	72% (0.60)	0.91 (0.64, 1.29)	VERY LOW
**Aged 13–18 years**				
Asthma incidence	2	0% (0.03)	**0.91 (0.83, 0.99)**	MODERATE
AR incidence	1	–	0.93 (0.76, 1.14)	–
**Aged 19–40 years**				
Asthma incidence	1	–	0.97 (0.85, 1.11)	–
AR incidence	1	–	0.99 (0.93, 1.05)	–
**Aged 40+ years**				
Asthma incidence	1	–	0.86 (0.77, 0.96)	–
COPD incidence/prevalence (aged 40–65 years)	3	91% (0.46)	0.97 (0.91, 1.05)	VERY LOW
COPD incidence/prevalence (aged over 65 years)	3	86% (0.16)	0.91 (0.79, 1.04)	VERY LOW

Weights are from random-effects analysis. Significant estimates are bolded. RR, relative risk; *I*^2^, heterogeneity statistic; CI, confidence interval; AR, allergic rhinitis; COPD, chronic obstructive pulmonary disease.

Ten of the 13 studies on the relationship between greenness exposure and asthma incidence/prevalence conducted analyses in different age subgroups. There are four studies for preschool children under 7 years old, two for school-aged children aged 8–12 years old, two studies for teenagers aged 13–18, one study for youth aged 18–40, and one study for people over 40. The estimated RRs for subgroups of 0–7 years, 8–12 years, and 13–18 years were 0.98 (95% CI: 0.80–1.20, *p* = 0.83, *I*^2^ = 51%), 0.91 (95% CI: 0.63–1.30, *p* = 0.60, *I*^2^ = 63%), and 0.91 (95% CI: 0.83–0.99, *p* = 0.03, *I*^2^ = 0%), respectively (Table 3 and Supplementary Figure S3). Moderate heterogeneity was observed for subgroups of 0–7 years and 8–12 years, and no heterogeneity was observed for 13- to 18-year subgroups.

Six of the seven studies on the relationship between greenness exposure and AR incidence/prevalence conducted analyses in different age subgroups. There are two studies for preschool children under seven, two for school-aged children aged 8–12 years old, one study for teenagers aged 13–18, and one study for youth aged 19–40. The estimated RRs for subgroups of 0–7 years and 8–12 years were 0.99 (95% CI: 0.83–1.19, *p* = 0.93, *I*^2^ = 0%) and 0.91 (95% CI: 0.64–1.29, *p* = 0.60, *I*^2^ = 72%), respectively (Table 3 and Supplementary Figure S4). Moderate heterogeneity emerged for 8- to 12-year subgroups, and no heterogeneity was observed for 0- to 7-year subgroups.

All four studies on the relationship between greenness exposure and COPD incidence/prevalence conducted analyses in different age subgroups. There are three studies for mid-life adults aged 40–65 and three studies for older adults aged over 65. The estimated RRs for subgroups of 40–65 years and over 65 years were 0.97 (95% CI: 0.91–1.05, *p* = 0.46, *I*^2^ = 91%) and 0.91 (95% CI: 0.79–1.04, *p* = 0.16, *I*^2^ = 86%), respectively (Table 3 and Supplementary Figure S5). High heterogeneity emerged for all the meta-analyses.

As a result of sensitivity analyses, we observed that most of the associations remained unchanged after any single study was excluded, which indicated that our findings were stable (Supplementary Table S11). According to the GRADE system, asthma incidence of 13–18 years had “moderate” confidence, AR incidence of 0–7 years had “low” confidence and other subgroups had “very low” confidence in the pooled evidence (Supplementary Table S7).

#### 3.3.2 Buffer subgroups

Considering the buffer zones of greenness exposure characteristics of the subjects in each study, we classified the buffer zones into three groups (200–300 m, 400–500 m, and 800–1,000 m buffers) (Table 4). All CRD outcomes had sufficient data to perform subgroup meta-analyses. Concrete study information is shown in Supplementary Figures S7–S12.

**Table 4:** Meta-analysis of buffer subgroups.

Outcomes	Studies (*n*)	Continuous exposure		GRADE
		Heterogeneity *I*^2^ (*p*-value)	Summary effect size (RR) (95% CI)	
**NDVI 200–300 m**				
Asthma incidence/prevalence	9	69% (0.02)	**0.92 (0.86, 0.98)**	VERY LOW
AR incidence/prevalence	5	69% (0.65)	1.02 (0.95, 1.09)	VERY LOW
COPD prevalence	1	–	1.05 (1.02, 1.08)	–
Lung cancer incidence	1	–	0.90 (0.77, 1.06)	–
Lung cancer mortality	5	80% (0.19)	0.98 (0.94, 1.01)	VERY LOW
COPD mortality	1	–	0.96 (0.90, 1.02)	–
**NDVI 400–500 m**				
Asthma incidence/prevalence	9	58% (0.09)	0.93 (0.85, 1.01)	VERY LOW
AR incidence/prevalence	7	0% (0.53)	0.99 (0.94, 1.03)	LOW
COPD incidence/prevalence	4	89% (0.18)	0.95 (0.89, 1.02)	VERY LOW
Lung cancer incidence	3	98% (0.09)	0.70 (0.46, 1.06)	VERY LOW
Lung cancer mortality	1	–	0.98 (0.97, 0.99)	–
**NDVI 800–1,000 m**				
Asthma incidence/prevalence	8	54% (<0.0001)	**0.87 (0.81, 0.93)**	VERY LOW
AR incidence/prevalence	4	26% (0.70)	0.99 (0.91, 1.06)	LOW
COPD incidence/prevalence	3	91% (0.13)	0.92 (0.83, 1.03)	VERY LOW
Lung cancer incidence	2	94% (0.31)	0.20 (0.01, 4.48)	VERY LOW
Lung cancer mortality	4	85% (0.43)	0.98 (0.94, 1.03)	VERY LOW
COPD mortality	2	0% (0.04)	**0.93 (0.88, 0.98)**	LOW

Weights are from random-effects analysis. Significant estimates are bolded. RR, relative risk; *I*^2^, heterogeneity statistic; CI, confidence interval; NDVI, normalized difference vegetation index; AR, allergic rhinitis; COPD, chronic obstructive pulmonary disease.

Ten of the 13 studies on the relationship between greenness exposure and asthma incidence/prevalence conducted analyses in different buffer subgroups. The estimated RRs were 0.92 (95% CI: 0.86–0.98, *p* = 0.02, *I*^2^ = 69% at 200–300 m buffer), 0.93 (95% CI: 085–1.01, *p* = 0.09, *I*^2^ = 58%) at 400–500 m buffer, and 0.87 (95% CI: 0.81–0.93, *p* < 0.0001, *I*^2^ = 54%) at 800–1000 m buffer, respectively (Table 4 and Supplementary Figure S7). Moderate heterogeneity emerged for all the meta-analyses.

All eight studies on the relationship between greenness exposure and AR incidence/prevalence conducted analyses in different buffer subgroups. The estimated RRs were 1.02 (95% CI: 0.95–1.09, *p* = 0.65, *I*^2^ = 69%) at 200–300 m buffer, 0.99 (95% CI: 0.94–1.03, *p* = 0.53, *I*^2^ = 0%) at 400–500 m buffer, and 0.99 (95% CI: 0.91–1.06, *p* = 0.70, *I*^2^ = 26%) at 800–1000 m buffer, respectively (Table 4 and Supplementary Figure S8). Moderate heterogeneity emerged in the NDVI 200–300 m subgroup, low heterogeneity was observed in the 800–1,000 m buffer subgroup, and no heterogeneity was found in the 400–500 m buffer subgroup.

All four studies on the relationship between greenness exposure and COPD incidence/prevalence conducted analyses in different buffer subgroups. The estimated RRs were 0.95 (95% CI: 0.89–1.02, *p* = 0.18, *I*^2^ = 89%) at 400–500 m buffer and 0.92 (95% CI: 0.83–1.03, *p* = 0.13, *I*^2^ = 91%) at 800–1000 m buffer (Table 4 and Supplementary Figure S9). High heterogeneity emerged for all the meta-analyses.

Four of the five studies on the relationship between greenness exposure and lung cancer incidence conducted analyses in different buffer subgroups. The estimated RRs for lung cancer incidence were 0.70 (95% CI: 0.46–1.06, *p* = 0.09; *I*^2^ = 98%) at 400–500 m buffer and 0.20 (95% CI: 0.01–4.48, *p* = 0.31, *I*^2^ = 94%) at 800–1000 m buffer, respectively (Table 4 and Supplementary Figure S10). High heterogeneity emerged for all the meta-analyses.

Five of the six studies on the relationship between greenness exposure and lung cancer mortality conducted analyses in different buffer subgroups. The estimated RRs were 0.98 (95% CI: 0.94–1.01, *p* = 0.19; *I*^2^ = 80%) at 200–300 m buffer and 0.98 (95% CI: 0.94–1.03, *p* = 0.43; *I*^2^ = 85%) at 800–1000 m buffer, respectively (Table 4 and Supplementary Figure S11). High heterogeneity emerged for all the meta-analyses.

All three studies on the relationship between greenness exposure and COPD mortality conducted analyses in different buffer subgroups. The estimated RR was 0.93 (95% CI: 0.88–0.98, *p* = 0.04, *I*^2^ = 0%) at 800–1000 m buffer (Table 4 and Supplementary Figure S12). No heterogeneity emerged for the meta-analyses.

As a result of sensitivity analyses, we observed that most of the associations remained unchanged after any single study was excluded, which indicated that our findings were stable (Supplementary Table S12). According to the GRADE system, AR incidence/prevalence at 400–500 m and 800–1,000 m buffers and COPD mortality at 800–1000 m buffer had “low” confidence; other subgroups had “very low” confidence in the pooled evidence (Supplementary Table S8).

### 3.4 Study RoB assessment

In Supplementary Tables S4, S5, we summarize the RoB valuations for studies exploring the connection between greenness exposure and chronic respiratory health issues. There was a single study that was deemed to have a “high risk of bias” (67). As far as confounding assessments and unblinded outcome assessments are concerned, all studies were deemed to have a “low risk of bias.” Nine studies were deemed to have a “high risk of bias” due to the bias in selecting the participants. Three studies were appraised to have a “high risk of bias” for health outcome assessment. Based on these indicators, no studies were excluded.

According to the funnel plot (Supplementary Figures S1, S2, S4, S11, S12), there was no evidence of published bias in this meta-analysis.

## 4 Discussion

This study identified 35 articles, consisting of presumably 32 million participants from 20 countries. A comprehensive evaluation was conducted according to the currently available data on the connection between greenness exposure and chronic respiratory health outcomes. Our study observed that the increment in greenness was connected with a reduction in asthma incidence, lung cancer incidence, and COPD mortality. Estimates of lung cancer incidence detected significant heterogeneity. The meta-analyses of age subgroups discovered that greenness exposure was connected with a low risk of asthma incidence in teenagers aged 13–18 years. A further meta-analysis of the buffer zone subgroups indicated that greenness exposure was positively correlated with asthma incidence/prevalence at 200–300 m and 800–1,000 m buffers, and COPD mortality was associated with greenness exposure at 800–1000 m buffers.

### 4.1 Comparison with other studies

Seven previously published reviews summarized the relationship between greenness and CRDs, which focused on one or two particular chronic respiratory health diseases (34). Our study assessed the relevance between greenness exposure and various CRD health outcomes. In line with this study, three previous meta-analyses indicated no significant overall association between greenness exposure and AR (33, 35, 37).

Three reviews and meta-analyses on asthma discovered no relationship between greenness exposure and asthma incidence (35–37) which is indirectly inconsistent with our findings (RR = 0.92, 95% CI: 0.85–0.98). Qiu et al. (34) combined 11 articles and reported asthma and rhinitis incidence and found that high-level residential greenness can significantly contribute to preventing respiratory diseases (OR = 0.95, 95% CI: 0.92–0.98). Our meta-analyses on different age subgroups observed that greenness exposure was correlated with a low risk of asthma incidence in teenagers aged 13–18 years (RR = 0.91, 95% CI: 0.83–0.99), but no connection was indicated in other age groups. According to Cao et al. (37) and Ye et al. (36), there is no significant correlation between greenness exposure and asthma in children and adolescents (aged under 19). It is thus clear that more detailed results for the age subgroup were provided in our study. Compared to children over 12 years old, Zeng et al. (54) found that greenness and asthma had a more positive relationship with children under 12 years old. In preschool years, Sbihi et al. (26) indicated a reduced asthma risk with every interquartile increase in NDVI (OR = 0.96, 95% CI: 0.93–0.99), but no similar relationships were observed during the school-age period. However, the finding was contrary to ours.

Our meta-analysis of the buffer subgroups concluded that greenness exposure was positively correlated with asthma incidence/prevalence at 200–300 m and 800–1,000 m buffers, and beneficial associations between greenness exposure at 800–1000 m buffer and asthma incidence/prevalence were stronger (RR = 0.87, 95% CI: 0.81–0.93). Inconsistent results were reported by a previous meta-analysis (35), which indicated no significant relationships between the NDVI and current asthma in the 0–100 m group, 100–300 m group, and 500–1,000 m group. Two cross-sectional studies found similar evidence to ours that stronger associations for greenness exposure in larger buffers (45, 54). The case–control study conducted in Kaunas confirmed the opposite finding that an IQR increment in NDVI-100 m was statistically significantly associated with an increase in asthma risk (OR = 1.43, 95% CI: 1.10–1.85) (48).

Our meta-analysis on lung cancer incidence and mortality was conducted separately, which indicated a significant positive correlation between greenness exposure and lung cancer incidence (RR = 0.62, 95% CI: 0.40–0.95). Sakhvidi et al. (39) combined nine studies on greenspace exposure and lung cancer incidence /mortality and observed no correlation between greenspace exposure and lung cancer outcomes. By contrast, we provided more detailed meta-analysis results.

In this study, we carried out the first meta-analysis of the connection between greenness exposure and COPD incidence/prevalence and mortality. We found that increased greenness exposure was related to a reduced risk of COPD mortality (RR = 0.95, 95% CI: 0.92–0.99), but not to COPD incidence and prevalence. However, three of four studies indicated the productive effect of greenness exposure on COPD (30, 53, 74). The higher prevalence of COPD has been linked to higher greenness levels across a countywide cross-sectional study (OR = 1.19, 95% CI: 1.13–1.27) (55). Possible reasons might be the method used to diagnose COPD, the different periods used to estimate NDVI, and the different dominant types of vegetation in different regions (55).

Greenness exposure was positively associated with COPD mortality at 800–1000 m buffer (RR = 0.93, 95% CI: 0.88–0.98) in our study. There is inconsistency in the study results included in our meta-analysis. An ecological study in Greece, consisting of 1,035 municipal units, reported that greenness with a 1,000 m buffer was protective for COPD mortality (86). A cohort study containing 14 sub-cohorts from Europe discovered no association between NDVI in 300 m and 1,000 m buffers and COPD mortality (49). Various types and populations of studies could contribute to the differences in estimate sizes across studies.

As compared to previously published reviews, our study specifically focuses on the role of greenness in various CRDs. To the best of our knowledge, this is the first study to explore quantitatively the relationship between chronic respiratory diseases among different ages. As a result, our review provides a deeper understanding of the epidemiologic relationships between greenness and CRDs than previous studies.

### 4.2 Potential mechanisms

**Figure 4:**
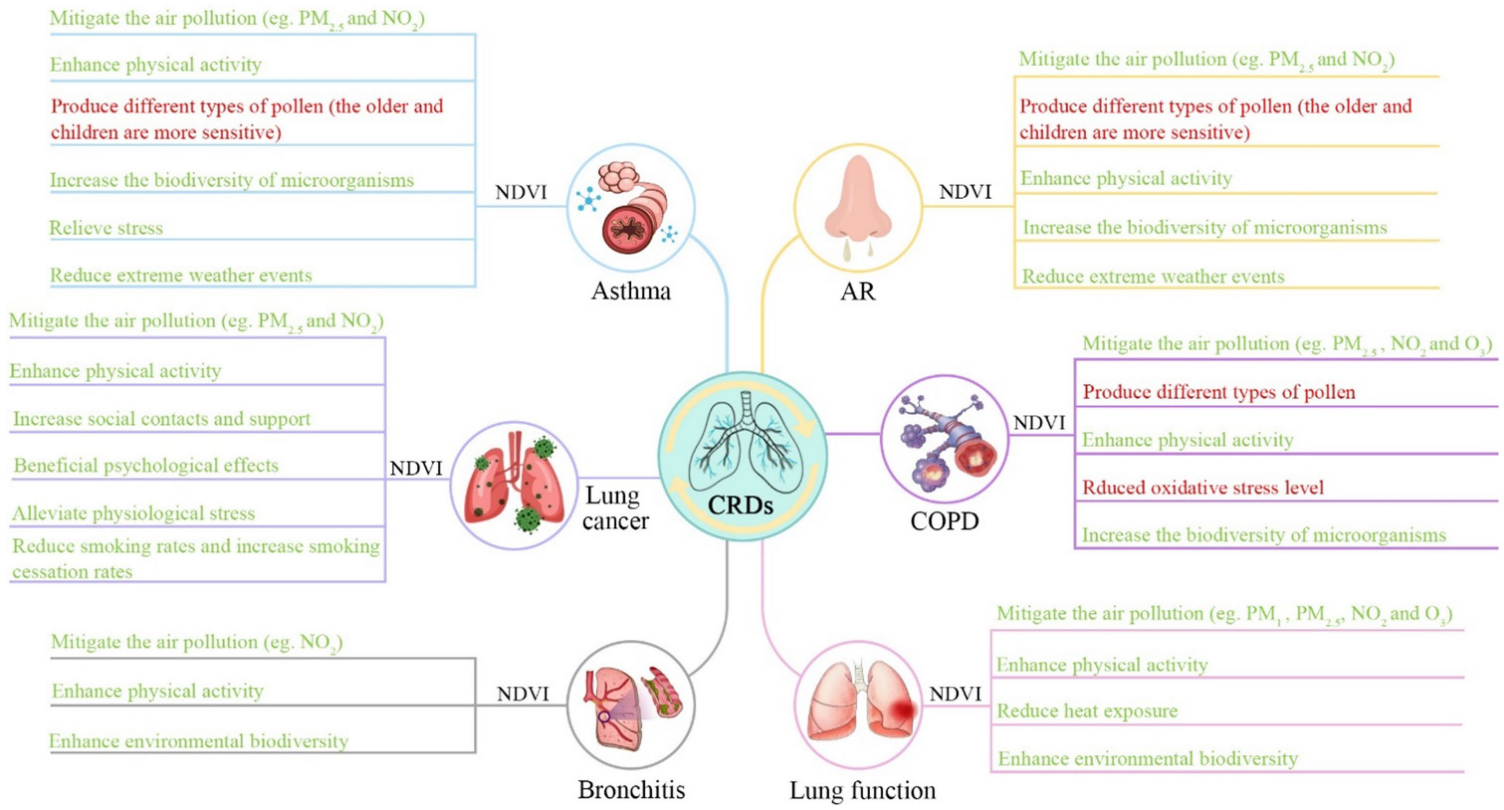
Potential mechanisms linking greenness exposure to chronic respiratory health outcomes. A number of factors in a green font may have a beneficial impact on respiratory health, whereas factors in a red font may have an adverse impact on their health.

Exposure to greenness remains unclear in terms of its mechanisms of action. The benefits of greenness on chronic respiratory health issues have been explained by several mechanisms. First, green vegetation can reduce heat exposure by absorbing solar radiation (89, 90). High temperatures are correlated with airway drying, which may trigger bronchoconstriction and affect respiratory health (91, 92). Second, greenness can attenuate air pollution. Based on the results of some studies, the most important symptoms of ozone (O_3_), particle matter (PM_10_), sulfide dioxide (SO_2_), nitrogen dioxide (NO_2_), polyaromatic hydrocarbons (PAHs), and metals increase respiratory morbidity and mortality (93–96). Vegetation and trees may reduce air pollution-related chronic respiratory health issues through dispersion, deposition, and modification of air pollutant concentrations (97, 98). Moreover, greenness can promote physical activity by providing places for play or exercise, as well as a place for walking or cycling (99). The evidence suggests that physical activity could serve as an elastic mechanism that can improve emotional regulation (100) and reduce stress reactivity (101). This can encourage well-being and reduce the odds of chronic respiratory health complications (102). Additionally, greenness can reduce inflammatory responses through exposure to a wider variety of microbes, which may contribute to the relationship between greenness and health (103). As a result of increased exposure to a more diverse microbial environment, immune tolerance may be enhanced, and CRDs may be prevented (104). The potential mechanisms of each specific CRD are integrated into Figure 4.

However, greenness may have problems with respiratory health as they are sources of emissions, VOC, molds, aerosols, and pollen, which have been indicated to create AR problems (105–108). Despite the fact that COPD is generally regarded as a non-allergic respiratory disease, some studies have observed that higher levels of pollen in green areas are connected with higher rates of hospitalization for COPD (76). This may be why our meta-analysis showed no significant association between AR and COPD incidence/prevalence.

Our study indicated that greenness exposure has a beneficial effect on asthma incidence in teenagers aged 13–18 years. As teenagers spend most of their time in school, inhalation rates are higher when they are engaged in medium- to intense physical activity compared to when they are sleeping at home or resting at home (109). The planting of roadside trees has been shown to reduce ambient levels of particulate matter, such as ultrafine particles (110), and decrease levels of heavy metals in PM2.5 (111). Thus, the downregulated air pollutants in the larger buffer zone from greenness exposure may be large enough to see a meaningful diminution in asthma risk. The relatively small buffer zone had a positive effect on asthma, which may be due to the visual impact of greenness on health seen from home (112), and living close to greener areas is likely to encourage more regular and active participation in physical activity (113).

In addition to age and buffer zones, there are also many potential factors related to greenness that can affect respiratory health, including atmospheric environment (71, 81), educational attainment of mothers (26), urban–rural differences (69, 70, 83), gender (85, 88), underweight or premature birth (25, 30, 81), and household income (25, 82). A more comprehensive analysis of different potential factors is necessary in the future, which may assist in reducing socio-economic health inequalities.

### 4.3 Strengths and limitations

Our study included comprehensive and up-to-date evidence on greenness exposure and various CRD incidence/prevalence and mortality. An important strength of our study was the assessment of greenness exposure across age groups and buffer groups with subgroup meta-analysis to explore associations with chronic respiratory health issues. Moreover, we evaluated the RoB and rated the quality of the evidence using the GRADE method to improve the credibility of the study.

However, our study also had a number of limitations. First, we were constrained by the number of studies. This makes it impossible to conduct meta-analyses on various types of CRDs, such as bronchiectasis and bronchitis. Second, the greenness exposure indicator we selected was NDVI, which cannot characterize the structure, quality, and accessibility of greenness. Third, studies with different potential confounders were included, and there was no uniformity among them. Thus, we cannot rule out confounding effects when estimating the effects of greenness exposure. Fourth, due to a lack of studies, a high degree of heterogeneity, and a RoB, it is difficult to draw any absolute conclusions about the relationship between greenness and CRDs. Furthermore, providing important estimates of impact, adjusting covariates as necessary, and explaining bias issues in detail will improve the quality of reports in the future. Fifth, pooling OR, HR, and RR results together could produce imprecise estimates of the influence of greenness exposure on chronic respiratory health outcomes. Finally, some subgroup analyses combine prevalence and incidence because of the limited number of studies, which may impair the quality of the results of a meta-analysis. Moreover, as a language restriction, we only included English-language manuscripts, which is also a limitation.

## 5 Conclusion

There was a correlation between higher greenness exposure and a reduced risk of some chronic respiratory health issues in this study, especially for asthma incidence, lung cancer incidence, and COPD mortality. Our results also indicate that age and buffer zone differences have potential implications for the correlation between greenness exposure and chronic respiratory health issues. According to this study, an increase in greenness exposure may be an appropriate measure to take precautions against some particular CRDs, potentially attracting the attention of policymakers and city managers to the public health issue. In order to provide detailed and specific recommendations for planners and policymakers, further research is necessary, focusing especially on the quality of greenness.

## Data availability statement

The original contributions presented in the study are included in the article/Supplementary material, further inquiries can be directed to the corresponding author.

## Author contributions

MT: Conceptualization, Data curation, Investigation, Software, Writing – original draft. WL: Conceptualization, Formal analysis, Methodology, Visualization, Writing – original draft. HL: Resources, Supervision, Validation, Visualization, Writing – review & editing. FL: Formal Analysis, Funding acquisition, Project administration, Resources, Supervision, Writing – review & editing.

## Glossary

CRD: chronic respiratory disease
DALY: disability-adjusted life-year
COPD: chronic obstructive pulmonary disease
AR: allergic rhinitis
CF: cystic fibrosis
NCFBE: non-cystic fibrosis bronchiectasis
IPF: idiopathic pulmonary fibrosis
UHI: urban heat island
NDVI: normalized difference vegetative index
PRISMA: Preferred Reporting Items for Systematic Reviews and Meta-Analyses
RoB: risk of bias
OR: odds ratio
RR: risk ratio
HR: hazard ratio
PM: particle matter
O_3_: Ozone
NO_2_: nitrogen dioxide
SO_2_: sulfide dioxide
PAH: polyaromatic hydrocarbon

## References

[1] Soriano JB Kendrick PJ Paulson KR Gupta V Abrams EM Adedoyin RA . Prevalence and attributable health burden of chronic respiratory diseases, 1990–2017: a systematic analysis for the global burden of disease study 2017. Lancet Respir Med. (2020) 8:585–96. doi: 10.1016/S2213-2600(20)30105-3, PMID: 32526187 PMC7284317

[2] Accordini S Corsico AG Calciano L Bono R Cerveri I Fois A . The impact of asthma, chronic bronchitis and allergic rhinitis on all-cause hospitalizations and limitations in daily activities: a population-based observational study. BMC Pulm Med. (2015) 15:10. doi: 10.1186/s12890-015-0008-0, PMID: 25880039 PMC4342897

[3] Mehta M Satija S Paudel KR Malyla V Kannaujiya VK Chellappan DK . Targeting respiratory diseases using miRNA inhibitor based nanotherapeutics: current status and future perspectives. Nanomedicine. (2021) 31:102303. doi: 10.1016/j.nano.2020.102303, PMID: 32980549

[4] Tan CL Chan Y Candasamy M Chellian J Madheswaran T Sakthivel LP . Unravelling the molecular mechanisms underlying chronic respiratory diseases for the development of novel therapeutics via in vitro experimental models. Eur J Pharmacol. (2022) 919:174821. doi: 10.1016/j.ejphar.2022.174821, PMID: 35151643

[5] The Lancet, GBD 2017: a fragile world. Lancet. (2018) 392:1683–3. doi: 10.1016/S0140-6736(18)32858-7, PMID: 30415747

[6] Li XC Cao XP Guo MZ Xie M Liu XS. Trends and risk factors of mortality and disability adjusted life years for chronic respiratory diseases from 1990 to 2017: systematic analysis for the global burden of disease study 2017. BMJ. (2020) 368:m234. doi: 10.1136/bmj.m234, PMID: 32075787 PMC7190065

[7] Eguiluz-Gracia I Mathioudakis AG Bartel S Vijverberg SJH Fuertes E Comberiati P . The need for clean air: the way air pollution and climate change affect allergic rhinitis and asthma. Allergy. (2020) 75:2170–84. doi: 10.1111/all.14177, PMID: 31916265

[8] Yuan M Song Y Huang Y Hong S Huang L. Exploring the association between urban form and air quality in China. J Plan Educ Res. (2017) 38:413–26. doi: 10.1177/0739456x17711516

[9] Kong L Lau KKL Yuan C Chen Y Xu Y Ren C . Regulation of outdoor thermal comfort by trees in Hong Kong. Sustain Cities Soc. (2017) 31:12–25. doi: 10.1016/j.scs.2017.01.018

[10] Yin CH Yuan M Lu YP Huang YP Liu YF. Effects of urban form on the urban heat island effect based on spatial regression model. Sci Total Environ. (2018) 634:696–704. doi: 10.1016/j.scitotenv.2018.03.350, PMID: 29649714

[11] Cardinale BJ Duffy JE Gonzalez A Hooper DU Perrings C Venail P . Biodiversity loss and its impact on humanity. Nature. (2012) 486:59–67. doi: 10.1038/nature11148 22678280

[12] Halonen JI Dehbi HM Hansell AL Gulliver J Fecht D Blangiardo M . Associations of night-time road traffic noise with carotid intima-media thickness and blood pressure: the Whitehall II and SABRE study cohorts. Environ Int. (2017) 98:54–61. doi: 10.1016/j.envint.2016.09.023, PMID: 27712935

[13] Wang VS Lo EW Liang CH Chao KP Bao BY Chang TY. Temporal and spatial variations in road traffic noise for different frequency components in metropolitan Taichung, Taiwan. Environ Pollut. (2016) 219:174–81. doi: 10.1016/j.envpol.2016.10.055, PMID: 27814533

[14] Deng Q Lu C Ou C Chen L Yuan H. Preconceptional, prenatal and postnatal exposure to outdoor and indoor environmental factors on allergic diseases/symptoms in preschool children. Chemosphere. (2016) 152:459–67. doi: 10.1016/j.chemosphere.2016.03.032, PMID: 27003368

[15] Bråbäck L Lodge CJ Lowe AJ Dharmage SC Olsson D Forsberg B. Childhood asthma and smoking exposures before conception—A three-generational cohort study. Pediatr Allergy Immunol. (2018) 29:361–8. doi: 10.1111/pai.12883, PMID: 29512835

[16] Weschler CJ. Changes in indoor pollutants since the 1950s. Atmos Environ. (2009) 43:153–69. doi: 10.1016/j.atmosenv.2008.09.044

[17] Lodge CJ Lowe AJ Gurrin LC Matheson MC Balloch A Axelrad C . Pets at birth do not increase allergic disease in at-risk children. Clin Exp Allergy. (2012) 42:1377–85. doi: 10.1111/j.1365-2222.2012.04032.x 22925324

[18] Zanini MJ Domínguez C Fernández-Oliva T Sánchez O Toda MT Foraster M . Urban-related environmental exposures during pregnancy and placental development and preeclampsia: a review. Curr Hypertens Rep. (2020) 22:81. doi: 10.1007/s11906-020-01088-4 32880755

[19] Franchini M Mannucci PM. Mitigation of air pollution by greenness: a narrative review. Eur J Intern Med. (2018) 55:1–5. doi: 10.1016/j.ejim.2018.06.021, PMID: 30180945

[20] Gisler A Korten I de Hoogh K Vienneau D Frey U Decrue F . Associations of air pollution and greenness with the nasal microbiota of healthy infants: a longitudinal study. Environ Res. (2021) 202:111633. doi: 10.1016/j.envres.2021.111633, PMID: 34256075

[21] Lu C Norback D Li YG Deng QH. Early-life exposure to air pollution and childhood allergic diseases: an update on the link and its implications. Expert Rev Clin Immunol. (2020) 16:813–27. doi: 10.1080/1744666X.2020.1804868, PMID: 32741235

[22] Mueller W Milner J Loh M Vardoulakis S Wilkinson P. Exposure to urban greenspace and pathways to respiratory health: an exploratory systematic review. Sci Total Environ. (2022) 829:154447. doi: 10.1016/j.scitotenv.2022.154447, PMID: 35283125

[23] Sun SZ Sarkar C Kumari S James P Cao W Lee RS . Air pollution associated respiratory mortality risk alleviated by residential greenness in the Chinese elderly Health service cohort. Environ Res. (2020) 183:109139. doi: 10.1016/j.envres.2020.109139, PMID: 31999997 PMC9847333

[24] Takaro TK Knowlton K Balmes JR. Climate change and respiratory health: current evidence and knowledge gaps. Expert Rev Respir Med. (2013) 7:349–61. doi: 10.1586/17476348.2013.814367, PMID: 23964626

[25] Donovan GH Gatziolis D Longley I Douwes J. Vegetation diversity protects against childhood asthma: results from a large New Zealand birth cohort. Nat Plants. (2018) 4:358-+. doi: 10.1038/s41477-018-0151-8, PMID: 29735984

[26] Sbihi H Tamburic L Koehoorn M Brauer M. Greenness and incident childhood asthma: a 10-year follow-up in a population-based birth cohort. Am J Respir Crit Care Med. (2015) 192:1131–3. doi: 10.1164/rccm.201504-0707LE, PMID: 26517419

[27] Su JG Barrett MA Henderson K Humblet O Smith T Sublett JW . Feasibility of deploying inhaler sensors to identify the impacts of environmental triggers and built environment factors on asthma short-acting bronchodilator use. Environ Health Perspect. (2017) 125:254–61. doi: 10.1289/EHP266, PMID: 27340894 PMC5289907

[28] Kim HJ Min JY Kim HJ Min KB. Association between green areas and allergic disease in Korean adults: a cross-sectional study. Ann Occup Environ Med. (2020) 32:e5. doi: 10.35371/aoem.2020.32.e5, PMID: 32082587 PMC7008584

[29] Kasdagli MI Katsouyanni K de Hoogh K Lagiou P Samoli E. Associations of air pollution and greenness with mortality in Greece: an ecological study. Environ Res. (2021) 196:110348. doi: 10.1016/j.envres.2020.110348, PMID: 33127394

[30] Sarkar C Zhang B Ni M Kumari S Bauermeister S Gallacher J . Environmental correlates of chronic obstructive pulmonary disease in 96 779 participants from the UK biobank: a cross-sectional, observational study. Lancet Planet Health. (2019) 3:E478–90. doi: 10.1016/S2542-5196(19)30214-1, PMID: 31777339

[31] Klompmaker JO Janssen NAH Bloemsma LD Marra M Lebret E Gehring U . Effects of exposure to surrounding green, air pollution and traffic noise with non-accidental and cause-specific mortality in the Dutch national cohort. Environ Health. (2021) 20:82. doi: 10.1186/s12940-021-00769-0, PMID: 34261495 PMC8281461

[32] Sun WY Bao PP Zhao XJ Tang J Wang L. Road traffic and urban form factors correlated with the incidence of Lung Cancer in high-density areas: an ecological study in downtown Shanghai, China. J Urban Health. (2021) 98:328–43. doi: 10.1007/s11524-021-00529-y, PMID: 33665783 PMC8190205

[33] Lambert KA Bowatte G Tham R Lodge C Prendergast L Heinrich J . Residential greenness and allergic respiratory diseases in children and adolescents – A systematic review and meta-analysis. Environ Res. (2017) 159:212–21. doi: 10.1016/j.envres.2017.08.002, PMID: 28803150

[34] Qiu Y Zuo SD Yu ZW Zhan Y Ren Y. Discovering the effects of integrated green space air regulation on human health: a bibliometric and meta-analysis. Ecol Indic. (2021) 132:108292. doi: 10.1016/j.ecolind.2021.108292

[35] Wu BR Guo X Liang M Sun C Gao J Xie P . Association of individual green space exposure with the incidence of asthma and allergic rhinitis: a systematic review and meta-analysis. Environ Sci Pollut Res. (2022) 29:88461–87. doi: 10.1007/s11356-022-23718-x, PMID: 36329245

[36] Ye TT Yu P Wen B Yang Z Huang W Guo Y . Greenspace and health outcomes in children and adolescents: a systematic review. Environ Pollut. (2022) 314:120193. doi: 10.1016/j.envpol.2022.120193, PMID: 36122655

[37] Cao NW Zhou HY du YJ Li XB Chu XJ Li BZ. The effect of greenness on allergic rhinitis outcomes in children and adolescents: a systematic review and meta-analysis. Sci Total Environ. (2023) 859:160244. doi: 10.1016/j.scitotenv.2022.160244, PMID: 36402344

[38] Parmes E Pesce G Sabel CE Baldacci S Bono R Brescianini S . Influence of residential land cover on childhood allergic and respiratory symptoms and diseases: evidence from 9 European cohorts. Environ Res. (2020) 183:108953. doi: 10.1016/j.envres.2019.108953, PMID: 31818476

[39] Sakhvidi MJZ Yang J Mehrparvar AH Dzhambov AM Ebrahimi A Dadvand P. Exposure to greenspace and cancer incidence, prevalence, and mortality: a systematic review and meta-analyses. Sci Total Environ. (2022) 838:156180. doi: 10.1016/j.scitotenv.2022.156180, PMID: 35618130

[40] Hussain MS Sharma P Dhanjal DS Khurana N Vyas M Sharma N . Nanotechnology based advanced therapeutic strategies for targeting interleukins in chronic respiratory diseases. Chem Biol Interact. (2021) 348:109637. doi: 10.1016/j.cbi.2021.109637, PMID: 34506765

[41] Wang Y Han X Li J Zhang L Liu Y Jin R . Associations between the compositional patterns of blood volatile organic compounds and chronic respiratory diseases and ages at onset in NHANES 2003–2012. Chemosphere. (2023) 327:138425:138425. doi: 10.1016/j.chemosphere.2023.138425, PMID: 36931402

[42] Liu X-X Ma XL Huang WZ Luo YN He CJ Zhong XM . Green space and cardiovascular disease: a systematic review with meta-analysis. Environ Pollut. (2022) 301:118990. doi: 10.1016/j.envpol.2022.118990, PMID: 35181451

[43] Su JG Jerrett M de Nazelle A Wolch J. Does exposure to air pollution in urban parks have socioeconomic, racial or ethnic gradients? Environ Res. (2011) 111:319–28. doi: 10.1016/j.envres.2011.01.002 21292252

[44] Wolch J Jerrett M Reynolds K McConnell R Chang R Dahmann N . Childhood obesity and proximity to urban parks and recreational resources: a longitudinal cohort study. Health Place. (2011) 17:207–14. doi: 10.1016/j.healthplace.2010.10.001, PMID: 21075670 PMC4380517

[45] Dadvand P Rivas I Basagaña X Alvarez-Pedrerol M Su J de Castro Pascual M . The association between greenness and traffic-related air pollution at schools. Sci Total Environ. (2015) 523:59–63. doi: 10.1016/j.scitotenv.2015.03.103 25862991

[46] Davies HW Vlaanderen JJ Henderson SB Brauer M. Correlation between co-exposures to noise and air pollution from traffic sources. Occup Environ Med. (2009) 66:347–50. doi: 10.1136/oem.2008.041764 19017692

[47] Zijlema WL Stasinska A Blake D Dirgawati M Flicker L Yeap BB . The longitudinal association between natural outdoor environments and mortality in 9218 older men from Perth, Western Australia. Environ Int. (2019) 125:430–6. doi: 10.1016/j.envint.2019.01.075, PMID: 30743148

[48] Andrusaityte S Grazuleviciene R Kudzyte J Bernotiene A Dedele A Nieuwenhuijsen MJ. Associations between neighbourhood greenness and asthma in preschool children in Kaunas, Lithuania: a case-control study. BMJ Open. (2016) 6:e010341. doi: 10.1136/bmjopen-2015-010341, PMID: 27067890 PMC4838715

[49] Bereziartua A Chen J de Hoogh K Rodopoulou S Andersen ZJ Bellander T . Exposure to surrounding greenness and natural-cause and cause-specific mortality in the ELAPSE pooled cohort. Environ Int. (2022) 166:107341. doi: 10.1016/j.envint.2022.107341, PMID: 35717714

[50] Dadvand P Villanueva CM Font-Ribera L Martinez D Basagaña X Belmonte J . Risks and benefits of green spaces for children: a cross-sectional study of associations with sedentary behavior, obesity, asthma, and allergy. Environ Health Perspect. (2014) 122:1329–35. doi: 10.1289/ehp.1308038, PMID: 25157960 PMC4256701

[51] Eldeirawi K Kunzweiler C Zenk S Finn P Nyenhuis S Rosenberg N . Associations of urban greenness with asthma and respiratory symptoms in Mexican American children. Ann Allergy Asthma Immunol. (2019) 122:289–95. doi: 10.1016/j.anai.2018.12.009, PMID: 30557617

[52] Rufo JC Paciencia I Hoffimann E Moreira A Barros H Ribeiro AI. The neighbourhood natural environment is associated with asthma in children: a birth cohort study. Allergy. (2021) 76:348–58. doi: 10.1111/all.14493, PMID: 32654186

[53] Xiao YL Gu X Niu H Meng X Zhang L Xu J . Associations of residential greenness with lung function and chronic obstructive pulmonary disease in China. Environ Res. (2022) 209:112877. doi: 10.1016/j.envres.2022.112877, PMID: 35131324

[54] Zeng XW Lowe AJ Lodge CJ Heinrich J Roponen M Jalava P . Greenness surrounding schools is associated with lower risk of asthma in schoolchildren. Environ Int. (2020) 143:105967. doi: 10.1016/j.envint.2020.105967, PMID: 32702595

[55] Fan J Guo Y Cao Z Cong S Wang N Lin H . Neighborhood greenness associated with chronic obstructive pulmonary disease: a nationwide cross-sectional study in China. Environ Int. (2020) 144:106042. doi: 10.1016/j.envint.2020.106042 32827808

[56] Page MJ McKenzie JE Bossuyt PM Boutron I Hoffmann TC Mulrow CD . The PRISMA 2020 statement: an updated guideline for reporting systematic reviews. Int J Surg. (2021) 88:105906. doi: 10.1016/j.ijsu.2021.105906, PMID: 33789826

[57] World Health Organization. WHO Handbook for Guideline Development [M/OL]. 2nd Edn. Geneva: World Health Organization, 2014. https://apps.who.int/iris/bitstream/handle/10665/145714/9789241548960_eng.pdf?sequence=1&isAllowed=y.

[58] van Kempen E Casas M Pershagen G Foraster M. WHO environmental noise guidelines for the European region: a systematic review on environmental noise and cardiovascular and metabolic effects: a summary. Int J Environ Res Public Health. (2018) 15:379. doi: 10.3390/ijerph15020379, PMID: 29470452 PMC5858448

[59] Langendam MW Akl EA Dahm P Glasziou P Guyatt G Schünemann HJ. Assessing and presenting summaries of evidence in Cochrane reviews. Syst Rev. (2013) 2:81. doi: 10.1186/2046-4053-2-81, PMID: 24059250 PMC3849859

[60] Welsh EJ Evans DJ Fowler SJ Spencer S. Interventions for bronchiectasis: an overview of Cochrane systematic reviews. Cochrane Database Syst Rev. (2015) 2015:CD010337. doi: 10.1002/14651858.CD010337.pub2, PMID: 26171905 PMC7086475

[61] Anderson HR Favarato G Atkinson RW. Long-term exposure to air pollution and the incidence of asthma: meta-analysis of cohort studies. Air Qual Atmos Health. (2013) 6:47–56. doi: 10.1007/s11869-011-0144-5

[62] Deeks J. When can odds ratios mislead? BMJ. (1998) 317:1155. doi: 10.1136/bmj.317.7166.1155a, PMID: 9784470 PMC1114127

[63] DerSimonian R Laird N. Meta-analysis in clinical trials revisited. Contemp Clin Trials. (2015) 45:139–45. doi: 10.1016/j.cct.2015.09.002, PMID: 26343745 PMC4639420

[64] Lin LF Chu H Hodges JS. Alternative measures of between-study heterogeneity in Meta-analysis: reducing the impact of outlying studies. Biometrics. (2017) 73:156–66. doi: 10.1111/biom.12543, PMID: 27167143 PMC5106349

[65] Higgins JP Thompson SG. Quantifying heterogeneity in a meta-analysis. Stat Med. (2002) 21:1539–58. doi: 10.1002/sim.1186, PMID: 12111919

[66] Gernes R Brokamp C Rice GE Wright JM Kondo MC Michael YL . Using high-resolution residential greenspace measures in an urban environment to assess risks of allergy outcomes in children. Sci Total Environ. (2019) 668:760–7. doi: 10.1016/j.scitotenv.2019.03.009, PMID: 30865906 PMC6563346

[67] Fuertes E Markevych I von Berg A Bauer CP Berdel D Koletzko S . Greenness and allergies: evidence of differential associations in two areas in Germany. J Epidemiol Community Health. (2014) 68:787–90. doi: 10.1136/jech-2014-203903, PMID: 24862831 PMC4112441

[68] Hartley K Ryan P Brokamp C Gillespie GL. Effect of greenness on asthma in children: a systematic review. Public Health Nurs. (2020) 37:453–60. doi: 10.1111/phn.12701, PMID: 31899558 PMC9292730

[69] Huang Y-J Lee PH Chen LC Lin BC Lin C Chan TC. Relationships among green space, ambient fine particulate matter, and cancer incidence in Taiwan: a 16-year retrospective cohort study. Environ Res. (2022) 212:113416. doi: 10.1016/j.envres.2022.113416, PMID: 35523280

[70] Kayyal-Tarabeia I Michael Y Lensky IM Blank M Agay-Shay K. Residential greenness and site-specific cancer: a registry based cohort of 144,427 participants with a 21-years of follow-up, Tel-Aviv district, Israel. Environ Res. (2022) 212:113460. doi: 10.1016/j.envres.2022.113460, PMID: 35561833

[71] Lee HY Wu YH Kusumaning Asri A Chen TH Pan WC Yu CP . Linkage between residential green spaces and allergic rhinitis among Asian children (case study: Taiwan). Landsc Urban Plan. (2020) 202:103868. doi: 10.1016/j.landurbplan.2020.103868

[72] Lin LZ Chen Y Wei J Wu S Wu S Jing J . The associations between residential greenness and allergic diseases in Chinese toddlers: a birth cohort study. Environ Res. (2022) 214:114003. doi: 10.1016/j.envres.2022.114003, PMID: 35931194

[73] Sakhvidi MJZ Yang J Siemiatycki J Dadvand P de Hoogh K Vienneau D . Greenspace exposure and cancer incidence: a 27-year follow-up of the French GAZEL cohort. Sci Total Environ. (2021) 787:147553. doi: 10.1016/j.scitotenv.2021.147553, PMID: 33989869

[74] Yu KX Zhang Q Meng X Zhang L Kan H Chen R. Association of residential greenness with incident chronic obstructive pulmonary disease: a prospective cohort study in the UK biobank. Environ Int. (2023) 171:107654. doi: 10.1016/j.envint.2022.107654, PMID: 36462434

[75] Dzhambov AM Lercher P Rudisser J Browning M Markevych I. Allergic symptoms in association with naturalness, greenness, and greyness: a cross-sectional study in schoolchildren in the Alps. Environ Res. (2021) 198:110456. doi: 10.1016/j.envres.2020.110456, PMID: 33188758

[76] Hanigan IC Johnston FH. Respiratory hospital admissions were associated with ambient airborne pollen in Darwin, Australia, 2004-2005. Clin Exp Allergy. (2007) 37:1556–65. doi: 10.1111/j.1365-2222.2007.02800.x, PMID: 17883735

[77] Li LY Hart J Coull B Cao SJ Spengler J Adamkiewicz G. Effect of residential greenness and nearby parks on respiratory and allergic diseases among middle school adolescents in a Chinese City. Int J Environ Res Public Health. (2019) 16:991. doi: 10.3390/ijerph16060991, PMID: 30893887 PMC6466062

[78] Yang L Yang Z Zhao Z Norbäck D Cai YS Zhang X. Exposure to greenness, air pollution and respiratory health among pre-school children in northern China. Atmos Environ. (2023) 298:119608. doi: 10.1016/j.atmosenv.2023.119608

[79] Nordeide Kuiper I Svanes C Markevych I Accordini S Bertelsen RJ Bråbäck L . Lifelong exposure to air pollution and greenness in relation to asthma, rhinitis and lung function in adulthood. Environ Int. (2021) 146:106219. doi: 10.1016/j.envint.2020.106219, PMID: 33126061

[80] Shao YQ Wang Y Yu H Zhang Y Xiang F Yang Y . Geographical variation in lung cancer risk associated with road traffics in Jiading District, Shanghai. Sci Total Environ. (2019) 652:729–35. doi: 10.1016/j.scitotenv.2018.10.266, PMID: 30380480

[81] Hu YB Chen Y Liu S Tan J Yu G Yan C . Residential greenspace and childhood asthma: an intra-city study. Sci Total Environ. (2023) 857:159792. doi: 10.1016/j.scitotenv.2022.159792, PMID: 36306842

[82] Wang JW Yang T Xu Z Jin J Wang Y Li G . Greenness and asthma in the middle-aged and elderly population in a prospective cohort study – China, 2011-2018. China CDC Wkly. (2022) 4:931–5. doi: 10.46234/ccdcw2022.191 PMC13423243 42535178

[83] Coleman CJ Yeager RA Riggs DW Coleman NC Garcia GR Bhatnagar A . Greenness, air pollution, and mortality risk: a US cohort study of cancer patients and survivors. Environ Int. (2021) 157:106797. doi: 10.1016/j.envint.2021.106797, PMID: 34332301

[84] Klompmaker JO Hoek G Bloemsma LD Marra M Wijga AH van den Brink C . Surrounding green, air pollution, traffic noise exposure and non-accidental and cause-specific mortality. Environ Int. (2020) 134:105341. doi: 10.1016/j.envint.2019.105341, PMID: 31783239

[85] Rodriguez-Loureiro L Verdoodt F Lefebvre W Vanpoucke C Casas L Gadeyne S. Long-term exposure to residential green spaces and site-specific cancer mortality in urban Belgium: a 13-year follow-up cohort study. Environ Int. (2022) 170:107571. doi: 10.1016/j.envint.2022.107571, PMID: 36219909

[86] Kasdagli MI Katsouyanni K de Hoogh K Lagiou P Samoli E. Investigating the association between long-term exposure to air pollution and greenness with mortality from neurological, cardio-metabolic and chronic obstructive pulmonary diseases in Greece. Environ Pollut. (2022) 292:118372. doi: 10.1016/j.envpol.2021.118372, PMID: 34656679

[87] Lee HY Wu CD Chang YT Chern YR Lung SCC Su HJ . Association between surrounding greenness and mortality: an ecological study in Taiwan. Int J Environ Res Public Health. (2020) 17:4525. doi: 10.3390/ijerph17124525, PMID: 32586013 PMC7344743

[88] Xu L Ren C Yuan C Nichol J Goggins W. An ecological study of the association between area-level green space and adult mortality in Hong Kong. Climate. (2017) 5:55. doi: 10.3390/cli5030055

[89] Li SS Baker PJ Jalaludin BB Marks GB Denison LS Williams GM. Ambient temperature and lung function in children with asthma in Australia. Eur Respir J. (2014) 43:1059–66. doi: 10.1183/09031936.00079313, PMID: 24311765

[90] Venter ZS Krog NH Barton DN. Linking green infrastructure to urban heat and human health risk mitigation in Oslo, Norway. Sci Total Environ. (2020) 709:136193. doi: 10.1016/j.scitotenv.2019.136193, PMID: 31887497

[91] Anderson SD Daviskas E. The mechanism of exercise-induced asthma is …. J Allergy Clin Immunol. (2000) 106:453–9. doi: 10.1067/mai.2000.109822 10984363

[92] Hales S Lewis S Slater T Crane J Pearce N. Prevalence of adult asthma symptoms in relation to climate in New Zealand. Environ Health Perspect. (1998) 106:607–10. doi: 10.1289/ehp.98106607, PMID: 9722625 PMC1533139

[93] Gryparis A Forsberg B Katsouyanni K Analitis A Touloumi G Schwartz J . Acute effects of ozone on mortality from the "air pollution and health: a European approach" project. Am J Respir Crit Care Med. (2004) 170:1080–7. doi: 10.1164/rccm.200403-333OC 15282198

[94] Kloog I Nordio F Zanobetti A Coull BA Koutrakis P Schwartz JD. Short term effects of particle exposure on hospital admissions in the mid-Atlantic states: a population estimate. PLoS One. (2014) 9:e88578. doi: 10.1371/journal.pone.0088578, PMID: 24516670 PMC3917892

[95] Norval M Lucas RM Cullen AP de Gruijl FR Longstreth J Takizawa Y . The human health effects of ozone depletion and interactions with climate change. Photochem Photobiol Sci. (2011) 10:199–225. doi: 10.1039/c0pp90044c, PMID: 21253670

[96] Pope CA 3rd Burnett RT Turner MC Cohen A Krewski D Jerrett M . Lung cancer and cardiovascular disease mortality associated with ambient air pollution and cigarette smoke: shape of the exposure-response relationships. Environ Health Perspect. (2011) 119:1616–21. doi: 10.1289/ehp.1103639, PMID: 21768054 PMC3226505

[97] Diener A Mudu P. How can vegetation protect us from air pollution? A critical review on green spaces' mitigation abilities for air-borne particles from a public health perspective – with implications for urban planning. Sci Total Environ. (2021) 796:148605. doi: 10.1016/j.scitotenv.2021.148605, PMID: 34271387

[98] van den Bosch M Nieuwenhuijsen M. No time to lose – green the cities now. Environ Int. (2017) 99:343–50. doi: 10.1016/j.envint.2016.11.025, PMID: 27923587

[99] Bedimo-Rung AL Mowen AJ Cohen DA. The significance of parks to physical activity and public health: a conceptual model. Am J Prev Med. (2005) 28:159–68. doi: 10.1016/j.amepre.2004.10.024 15694524

[100] Chan JSY Liu G Liang D Deng K Wu J Yan JH. Special issue – therapeutic benefits of physical activity for mood: a systematic review on the effects of exercise intensity, duration, and modality. J Psychol. (2019) 153:102–25. doi: 10.1080/00223980.2018.1470487, PMID: 30321106

[101] Mücke M Ludyga S Colledge F Gerber M. Influence of regular physical activity and fitness on stress reactivity as measured with the Trier social stress test protocol: a systematic review. Sports Med. (2018) 48:2607–22. doi: 10.1007/s40279-018-0979-0, PMID: 30159718

[102] Belcher BR Zink J Azad A Campbell CE Chakravartti SP Herting MM. The roles of physical activity, exercise, and fitness in promoting resilience during adolescence: effects on mental well-being and brain development. Biol Psychiatry Cogn Neurosci Neuroimaging. (2021) 6:225–37. doi: 10.1016/j.bpsc.2020.08.005, PMID: 33067166 PMC7878276

[103] Rook GA. Regulation of the immune system by biodiversity from the natural environment: an ecosystem service essential to health. Proc Natl Acad Sci U S A. (2013) 110:18360–7. doi: 10.1073/pnas.1313731110, PMID: 24154724 PMC3831972

[104] von Mutius E. The microbial environment and its influence on asthma prevention in early life. J Allergy Clin Immunol. (2016) 137:680–9. doi: 10.1016/j.jaci.2015.12.1301, PMID: 26806048

[105] Gibbs JEM. Essential oils, asthma, thunderstorms, and plant gases: a prospective study of respiratory response to ambient biogenic volatile organic compounds (BVOCs). J Asthma Allergy. (2019) 12:169–182. doi: 10.2147/JAA.S193211 31417289 PMC6593190

[106] Cecchi L D'Amato G Annesi-Maesano I. External exposome and allergic respiratory and skin diseases. J Allergy Clin Immunol. (2018) 141:846–57. doi: 10.1016/j.jaci.2018.01.016, PMID: 29519451

[107] Marchetti P Pesce G Villani S Antonicelli L Ariano R Attena F . Pollen concentrations and prevalence of asthma and allergic rhinitis in Italy: evidence from the GEIRD study. Sci Total Environ. (2017) 584–585:1093–9. doi: 10.1016/j.scitotenv.2017.01.168, PMID: 28169023

[108] Schuler Iv CF Montejo JM. Allergic rhinitis in children and adolescents. Pediatr Clin N Am. (2019) 66:981–93. doi: 10.1016/j.pcl.2019.06.004 31466686

[109] Ma S Xu C Ma J Wang Z Zhang Y Shu Y . Association between perfluoroalkyl substance concentrations and blood pressure in adolescents. Environ Pollut. (2019) 254:112971. doi: 10.1016/j.envpol.2019.112971, PMID: 31394346

[110] Hagler GSW Lin M-Y Khlystov A Baldauf RW Isakov V Faircloth J . Field investigation of roadside vegetative and structural barrier impact on near-road ultrafine particle concentrations under a variety of wind conditions. Sci Total Environ. (2012) 419:7–15. doi: 10.1016/j.scitotenv.2011.12.002, PMID: 22281040

[111] Ozdemir H. Mitigation impact of roadside trees on fine particle pollution. Sci Total Environ. (2019) 659:1176–85. doi: 10.1016/j.scitotenv.2018.12.262, PMID: 31096331

[112] Su JG Dadvand P Nieuwenhuijsen MJ Bartoll X Jerrett M. Associations of green space metrics with health and behavior outcomes at different buffer sizes and remote sensing sensor resolutions. Environ Int. (2019) 126:162–70. doi: 10.1016/j.envint.2019.02.008, PMID: 30798197

[113] Lachowycz K Jones AP. Greenspace and obesity: a systematic review of the evidence. Obes Rev. (2011) 12:e183–9. doi: 10.1111/j.1467-789X.2010.00827.x, PMID: 21348919

