# Supplementary material for: Greenness and chronic respiratory health issues: a systematic review and meta-analysis
Source: Front Public Health. 2023 Dec 6;11:1279322. doi: 10.3389/fpubh.2023.1279322 (PMC10732026; doi:10.3389/fpubh.2023.1279322)
Supplement: Supplementary file 1 [file Data_Sheet_1.PDF]

Table S1. Supplemental Preferred Reporting Items for Systematic reviews and Meta-Analysis (PRISMA) 2020 Checklist

Section and Topic	Item #	Checklist item	Location where item is reported
**TITLE**			
Title	1	Identify the report as a systematic review.	1
**ABSTRACT**			
Abstract	2	See the PRISMA 2020 for Abstracts checklist.	1
**INTRODUCTION**			
Rationale	3	Describe the rationale for the review in the context of existing knowledge.	2
Objectives	4	Provide an explicit statement of the objective(s) or question(s) the review addresses.	4
**METHODS**			
Eligibility criteria	5	Specify the inclusion and exclusion criteria for the review and how studies were grouped for the syntheses.	4
Information sources	6	Specify all databases, registers, websites, organisations, reference lists and other sources searched or consulted to identify studies. Specify the date when each source was last searched or consulted.	4
Search strategy	7	Present the full search strategies for all databases, registers and websites, including any filters and limits used.	4
Selection process	8	Specify the methods used to decide whether a study met the inclusion criteria of the review, including how many reviewers screened each record and each report retrieved, whether they worked independently, and if applicable, details of automation tools used in the process.	4-5
Data collection process	9	Specify the methods used to collect data from reports, including how many reviewers collected data from each report, whether they worked independently, any processes for obtaining or confirming data from study investigators, and if applicable, details of automation tools used in the process.	5
Data items	10a	List and define all outcomes for which data were sought. Specify whether all results that were compatible with each outcome	5

Section and Topic	Item #	Checklist item	Location where item is reported
		domain in each study were sought (e.g. for all measures, time points, analyses), and if not, the methods used to decide which results to collect.	
	10b	List and define all other variables for which data were sought (e.g. participant and intervention characteristics, funding sources). Describe any assumptions made about any missing or unclear information.	NA
Study risk of bias assessment	11	Specify the methods used to assess risk of bias in the included studies, including details of the tool(s) used, how many reviewers assessed each study and whether they worked independently, and if applicable, details of automation tools used in the process.	4-5
Effect measures	12	Specify for each outcome the effect measure(s) (e.g. risk ratio, mean difference) used in the synthesis or presentation of results.	5
Synthesis methods	13a	Describe the processes used to decide which studies were eligible for each synthesis (e.g. tabulating the study intervention characteristics and comparing against the planned groups for each synthesis (item #5)).	4
	13b	Describe any methods required to prepare the data for presentation or synthesis, such as handling of missing summary statistics, or data conversions.	5
	13c	Describe any methods used to tabulate or visually display results of individual studies and syntheses.	5
	13d	Describe any methods used to synthesize results and provide a rationale for the choice(s). If meta-analysis was performed, describe the model(s), method(s) to identify the presence and extent of statistical heterogeneity, and software package(s) used.	5
	13e	Describe any methods used to explore possible causes of heterogeneity among study results (e.g. subgroup analysis, meta-regression).	5
	13f	Describe any sensitivity analyses conducted to assess robustness of the synthesized results.	5
Reporting bias	14	Describe any methods used to assess risk of bias due to missing results in a synthesis (arising from reporting biases).	5

Section and Topic	Item #	Checklist item	Location where item is reported
assessment			
Certainty assessment	15	Describe any methods used to assess certainty (or confidence) in the body of evidence for an outcome.	4-5
**RESULTS**			
Study selection	16a	Describe the results of the search and selection process, from the number of records identified in the search to the number of studies included in the review, ideally using a flow diagram.	5-6
	16b	Cite studies that might appear to meet the inclusion criteria, but which were excluded, and explain why they were excluded.	5-6
Study characteristics	17	Cite each included study and present its characteristics.	6-7; 8
Risk of bias in studies	18	Present assessments of risk of bias for each included study.	11
Results of individual studies	19	For all outcomes, present, for each study: (a) summary statistics for each group (where appropriate) and (b) an effect estimate and its precision (e.g. confidence/credible interval), ideally using structured tables or plots.	7-11
Results of syntheses	20a	For each synthesis, briefly summarise the characteristics and risk of bias among contributing studies.	7-11
	20b	Present results of all statistical syntheses conducted. If meta-analysis was done, present for each the summary estimate and its precision (e.g. confidence/credible interval) and measures of statistical heterogeneity. If comparing groups, describe the direction of the effect.	7-11
	20c	Present results of all investigations of possible causes of heterogeneity among study results.	7-11
	20d	Present results of all sensitivity analyses conducted to assess the robustness of the synthesized results.	7-11
Reporting biases	21	Present assessments of risk of bias due to missing results (arising from reporting biases) for each synthesis assessed.	NA

Section and Topic	Item #	Checklist item	Location where item is reported
Certainty of evidence	22	Present assessments of certainty (or confidence) in the body of evidence for each outcome assessed.	7-11
**DISCUSSION**			
Discussion	23a	Provide a general interpretation of the results in the context of other evidence.	13-14
	23b	Discuss any limitations of the evidence included in the review.	15
	23c	Discuss any limitations of the review processes used.	15
	23d	Discuss implications of the results for practice, policy, and future research.	15
**OTHER INFORMATION**			
Registration and protocol	24a	Provide registration information for the review, including register name and registration number, or state that the review was not registered.	12-13
	24b	Indicate where the review protocol can be accessed, or state that a protocol was not prepared.	NA
	24c	Describe and explain any amendments to information provided at registration or in the protocol.	NA
Support	25	Describe sources of financial or non-financial support for the review, and the role of the funders or sponsors in the review.	15
Competing interests	26	Declare any competing interests of review authors.	15
Availability of data, code and other materials	27	Report which of the following are publicly available and where they can be found: template data collection forms; data extracted from included studies; data used for all analyses; analytic code; any other materials used in the review.	NA

From: Page, M.J., McKenzie, J.E., Bossuyt, P.M., Boutron, I., Hoffmann, T.C., Mulrow, C.D., et al., 2021. The PRISMA 2020 statement: an updated guideline

for reporting systematic reviews. BMJ. 372, n71.

Table S2. Studies search strategy (up until December 31, 2022)

Database	Search strategies	Results
PubMed	(Green space[Title/Abstract] OR Greenspace[Title/Abstract] OR Greenness[Title/Abstract] OR Greenery[Title/Abstract] OR Garden[Title/Abstract] OR NDVI[Title/Abstract] OR Normalized Difference Vegetation Index[Title/Abstract] OR Wild land [Title/Abstract] OR Wild area[Title/Abstract] OR Wilderness[Title/Abstract] OR natural area[Title/Abstract] OR Natural lan d[Title/Abstract] OR Municipal land[Title/Abstract] OR recreation resource[Title/Abstract] OR natural amenities[Title/Abstra ct] OR Community land[Title/Abstract] OR Public land[Title/Abstract] OR Open land[Title/Abstract] OR Open space[Title/ Abstract] OR Natural environment[Title/Abstract] OR Urban design[Title/Abstract] OR Built environment[Title/Abstract] O R Park[Title/Abstract] OR Forest[Title/Abstract] OR Residential green[Title/Abstract] OR Tree canopy[Title/Abstract] OR T ree cover[Title/Abstract] OR Vegetation[Title/Abstract] OR Leaf area index[Title/Abstract] OR Greenbelt[Title/Abstract] OR Green area[Title/Abstract] OR Botanic park[Title/Abstract] OR City park[Title/Abstract] OR Public garden[Title/Abstract] OR Park availability[Title/Abstract] OR Natural neighborhood[Title/Abstract] OR Natural facilities[Title/Abstract] OR Cycle path[Title/Abstract] OR Park access[Title/Abstract] OR wild space[Title/Abstract] OR public space[Title/Abstract] OR natu ral space[Title/Abstract] OR natural place[Title/Abstract] OR community space[Title/Abstract] OR municipal space[Title/Abs tract] OR path green[Title/Abstract] OR trail green[Title/Abstract] OR greenbelt[Title/Abstract] OR neighborhood/neighborh ood environment[Title/Abstract] OR living environment[Title/Abstract] OR residential environment[Title/Abstract] OR enviro nmental feature[Title/Abstract] OR physical environment[Title/Abstract] OR physical activity resource[Title/Abstract] OR re creation opportunities[Title/Abstract] OR recreation destination[Title/Abstract] OR recreation facilities[Title/Abstract]) AND (Respiratory Diseases[Title/Abstract] OR Disease, Respiratory Tract[Title/Abstract] OR Respiratory Tract Disease[Title/Abstr act] OR Respiratory System Diseases[Title/Abstract] OR Disease, Respiratory System[Title/Abstract] OR Respiratory Syste m Disease[Title/Abstract] OR acute respiratory tract disease[Title/Abstract] OR adult respiratory distress syndrome[Title/Ab stract] OR respiratory tract allergy[Title/Abstract] OR chronic aspecific respiratory tract disease[Title/Abstract] OR experim ental respiratory disease[Title/Abstract] OR Asthma[Title/Abstract] OR Bronchial Asthma[Title/Abstract] OR Asthma, Bronc hial[Title/Abstract] OR Asthma-Chronic Obstructive Pulmonary Disease Overlap Syndrome[Title/Abstract] OR Asthma, Aspi	2593

	rin-Induced[Title/Abstract] OR Asthma, Exercise-Induced[Title/Abstract] OR Asthma, Occupational[Title/Abstract] OR Status Asthmaticus[Title/Abstract] OR allergic asthma[Title/Abstract] OR asthmatic state[Title/Abstract] OR extrinsic asthma[Title/Abstract] OR intrinsic asthma[Title/Abstract] OR nocturnal asthma[Title/Abstract] OR Chronic Obstructive Lung Disease[Title/Abstract] OR Chronic Obstructive Pulmonary Diseases[Title/Abstract] OR COAD[Title/Abstract] OR COPD[Title/Abstract] OR Chronic Obstructive Airway Disease[Title/Abstract] OR Chronic Obstructive Pulmonary Disease[Title/Abstract] OR Airflow Obstruction, Chronic[Title/Abstract] OR Airflow Obstructions, Chronic[Title/Abstract] OR Chronic Airflow Obstructions[Title/Abstract] OR Chronic Airflow Obstruction[Title/Abstract] OR Rhinitis[Title/Abstract] OR allergic rhinitis[Title/Abstract] OR perennial rhinitis[Title/Abstract] OR pollen allergy[Title/Abstract] OR atrophic rhinitis[Title/Abstract] OR chronic rhinitis[Title/Abstract] OR common cold[Title/Abstract] OR experimental rhinitis[Title/Abstract] OR rhinoconjunctivitis[Title/Abstract] OR rhinopharyngitis[Title/Abstract] OR rhinosinusitis[Title/Abstract] OR vasomotor rhinitis[Title/Abstract] OR respiratory symptoms[Title/Abstract] OR Apnea[Title/Abstract] OR Brief, Resolved, Unexplained Event[Title/Abstract] OR Cheyne-Stokes Respiration[Title/Abstract] OR Cough[Title/Abstract] OR Dyspnea[Title/Abstract] OR Epistaxis[Title/Abstract] OR Hemoptysis[Title/Abstract] OR Hoarseness[Title/Abstract] OR Hypercapnia[Title/Abstract] OR Hyperoxia[Title/Abstract] OR Hyperventilation[Title/Abstract] OR Hypocapnia[Title/Abstract] OR Hypoventilation[Title/Abstract] OR Hypoxia[Title/Abstract] OR Mouth Breathing[Title/Abstract] OR Respiratory Sounds[Title/Abstract] OR Rhinorrhea[Title/Abstract] OR Sneezing[Title/Abstract] OR Tachypnea[Title/Abstract] OR wheezing[Title/Abstract] OR lung cancer[Title/Abstract] OR Lung Neoplasms[Title/Abstract])	
Web of Science	((TI=(Green space OR Greenspace OR Greenness OR Greenery OR Garden OR NDVI OR Normalized Difference Vegetation Index OR Wild land OR Wild area OR Wilderness OR natural area OR Natural land OR Municipal land OR recreation resource OR natural amenities OR Community land OR Public land OR Open land OR Open space OR Natural environment OR Urban design OR Built environment OR Park OR Forest OR Residential green OR Tree canopy OR Tree cover OR Vegetation OR Leaf area index OR Greenbelt OR Green area OR Botanic park OR City park OR Public garden OR Park availability OR Natural neighborhood OR Natural facilities OR Cycle path OR Park access OR wild space OR public space OR natural space OR natural place OR community space OR municipal space OR path green OR trail green OR greenbelt OR neighborhood/neighborhood environment OR living environment OR residential environment OR environmental feature OR physical environment OR physical activity resource OR recreation opportunities OR recreation des	171

	tination OR recreation facilities))) AND TI=(Respiratory Diseases OR Disease, Respiratory Tract OR Respiratory Tract Disease OR Respiratory Diseases OR Respiratory System Diseases OR Disease, Respiratory System OR Respiratory System Disease OR acute respiratory tract disease OR adult respiratory distress syndrome OR respiratory tract allergy OR chronic aspecific respiratory tract disease OR experimental respiratory disease OR Asthma OR Bronchial Asthma OR Asthma, Bronchial OR Asthma-Chronic Obstructive Pulmonary Disease Overlap Syndrome OR Asthma, Aspirin-Induced OR Asthma, Exercise-Induced OR Asthma, Occupational OR Status Asthmaticus OR allergic asthma OR asthmatic state OR extrinsic asthma OR intrinsic asthma OR nocturnal asthma OR Chronic Obstructive Lung Disease OR Chronic Obstructive Pulmonary Diseases OR COAD OR COPD OR Chronic Obstructive Airway Disease OR Chronic Obstructive Pulmonary Disease OR Airflow Obstruction, Chronic OR Airflow Obstructions, Chronic OR Chronic Airflow Obstructions OR Chronic Airflow Obstruction OR Rhinitis OR allergic rhinitis OR perennial rhinitis OR pollen allergy OR atrophic rhinitis OR chronic rhinitis OR common cold OR experimental rhinitis OR rhinoconjunctivitis OR rhinopharingitis OR rhinosinusitis OR vasomotor rhinitis OR respiratory symptoms OR Apnea OR Brief, Resolved, Unexplained Event OR Cheyne-Stokes Respiration OR Cough OR Dyspnea OR Epistaxis OR Hemoptysis OR Hoarseness OR Hypercapnia OR Hyperoxia OR Hyperventilation OR Hypocapnia OR Hypoventilation OR Hypoxia OR Mouth Breathing OR Respiratory Sounds OR Rhinorrhea OR Sneezing OR Tachypnea OR wheezing OR lung cancer OR Lung Neoplasms)	
Embase	('green space':ab,ti OR 'greenspace':ab,ti OR 'greenness':ab,ti OR 'greenery':ab,ti OR 'garden':ab,ti OR 'ndvi':ab,ti OR 'normalized difference vegetation index':ab,ti OR 'wild land':ab,ti OR 'wild area':ab,ti OR 'wilderness':ab,ti OR 'natural area':ab,ti OR 'natural land':ab,ti OR 'municipal land':ab,ti OR 'recreation resource':ab,ti OR 'natural amenities':ab,ti OR 'community land':ab,ti OR 'public land':ab,ti OR 'open land':ab,ti OR 'open space':ab,ti OR 'natural environment':ab,ti OR 'urban design':ab,ti OR 'built environment':ab,ti OR 'park':ab,ti OR 'forest':ab,ti OR 'residential green':ab,ti OR 'tree canopy':ab,ti OR 'tree cover':ab,ti OR 'vegetation':ab,ti OR 'leaf area index':ab,ti OR 'green area':ab,ti OR 'botanic park':ab,ti OR 'city park':ab,ti OR 'public garden':ab,ti OR 'park availability':ab,ti OR 'natural neighborhood':ab,ti OR 'natural facilities':ab,ti OR 'cycle path':ab,ti OR 'park access':ab,ti OR 'wild space':ab,ti OR 'public space':ab,ti OR 'natural space':ab,ti OR 'natural place':ab,ti OR 'community space':ab,ti OR 'municipal space':ab,ti OR 'path green':ab,ti OR 'trail green':ab,ti OR 'greenbelt':ab,ti OR 'neighborhood/neighborhood environment':ab,ti OR 'living environment':ab,ti OR 'residential environment':ab,ti OR 'environmental feature':ab,ti OR 'physical environment':ab,ti OR 'physical activity resource':ab,ti OR 'recreation opportunities':ab,ti OR 'recreation destination':ab,t	3927

	i OR 'recreation facilities':ab,ti) AND ('disease, respiratory tract':ab,ti OR 'respiratory tract disease':ab,ti OR 'respiratory diseases':ab,ti OR 'respiratory system diseases':ab,ti OR 'disease, respiratory system':ab,ti OR 'respiratory system disease':ab,ti OR 'acute respiratory tract disease':ab,ti OR 'adult respiratory distress syndrome':ab,ti OR 'respiratory tract allergy':ab,ti OR 'chronic aspecific respiratory tract disease':ab,ti OR 'experimental respiratory disease':ab,ti OR 'asthma':ab,ti OR 'bronchial asthma':ab,ti OR 'asthma, bronchial':ab,ti OR 'asthma-chronic obstructive pulmonary disease overlap syndrome':ab,ti OR 'asthma, aspirin-induced':ab,ti OR 'asthma, exercise-induced':ab,ti OR 'asthma, occupational':ab,ti OR 'status asthmaticus':ab,ti OR 'allergic asthma':ab,ti OR 'asthmatic state':ab,ti OR 'extrinsic asthma':ab,ti OR 'intrinsic asthma':ab,ti OR 'nocturnal asthma':ab,ti OR 'chronic obstructive lung disease':ab,ti OR 'chronic obstructive pulmonary diseases':ab,ti OR 'coad':ab,ti OR 'copd':ab,ti OR 'chronic obstructive airway disease':ab,ti OR 'chronic obstructive pulmonary disease':ab,ti OR 'airflow obstruction, chronic':ab,ti OR 'airflow obstructions, chronic':ab,ti OR 'chronic airflow obstructions':ab,ti OR 'chronic airflow obstruction':ab,ti OR 'rhinitis':ab,ti OR 'allergic rhinitis':ab,ti OR 'perennial rhinitis':ab,ti OR 'pollen allergy':ab,ti OR 'atrophic rhinitis':ab,ti OR 'chronic rhinitis':ab,ti OR 'common cold':ab,ti OR 'experimental rhinitis':ab,ti OR 'rhinoconjunctivitis':ab,ti OR 'rhinopharyngitis':ab,ti OR 'rhinosinusitis':ab,ti OR 'vasomotor rhinitis':ab,ti OR 'respiratory symptoms':ab,ti OR 'apnea':ab,ti OR 'brief, resolved, unexplained event':ab,ti OR 'cheyne-stokes respiration':ab,ti OR 'cough':ab,ti OR 'dyspnea':ab,ti OR 'epistaxis':ab,ti OR 'hemoptysis':ab,ti OR 'hoarseness':ab,ti OR 'hypercapnia':ab,ti OR 'hyperoxia':ab,ti OR 'hyperventilation':ab,ti OR 'hypocapnia':ab,ti OR 'hypoventilation':ab,ti OR 'hypoxia':ab,ti OR 'mouth breathing':ab,ti OR 'respiratory sounds':ab,ti OR 'rhinorrhea':ab,ti OR 'sneezing':ab,ti OR 'tachypnea':ab,ti OR 'wheezing':ab,ti OR 'lung cancer':ab,ti OR 'Lung Neoplasms':ab,ti)	

Table S3. Descriptive summaries of all included studies

Study	Country	Study design	Timeframe	Sample size(n)	Maternal age(year)	NDVI Buffer size(m)	NDVI Exposure unit	Outcomes	Confounders/covariates
**Odd Ratio (OR)**									
Andrusaityte et al.,2016	Konas, Lithuania	Case-control	2012-2013	1489	0-6y	100/300/500	Per IQR	Asthma incidence	Parental asthma, maternal education, childbirth age, smoked during pregnancy, breastfeeding, antibiotics in the first year, cats in the past 12 months, living in apartments, spending time in green space
Rufo et al., 2021	Porto, Portugal	Cohort	2005-2012	1050	0-7y	100/200/300	Per 0.1	Asthma prevalence	Gender, community deprivation, distance from main roads, mother's history of asthma, congestion, and mother's education
Dadvand et al., 2014	Sabadell, Spain	Cross-sectional	2006	3178	9-12y	100/250/500/1000	Per IQR	Asthma and allergic rhino-conjunctivitis prevalence	Child's sex and age, exposure to environmental tobacco smoke at home, having older siblings, type of school (public vs. private), parental education, and parental history of asthma
Donovan et al., 2018	New Zealand	Cohort	1998-2016	49956	0-18y	mean buffer ~255	Per 0.1	Asthma prevalence	Air pollution (major road length, mean annual NO_2), premature birth, low birth weight, antibiotic use, parental smoking, ethnicity, birth order, number of siblings, parental occupation, NZDep social deprivation index
Dzhambov et al., 2021	Alpine towns, Austria & Italy	Cross-sectional	2004/2005	1251	8-12y	100/500/1000	Per IQR	Asthma and AR prevalence	Age, gender, mother's education, low birth weight, smoking during pregnancy, breastfeeding time, the cumulative risk of second-hand smoke/pneumonia/bronchitis in the first year, number of green months during pregnancy, geographical area
Eldeirawi et al., 2019	Chicago	Cross-sectional	2004.08-2005.04	1915	4-18y	100/250/500	Per IQR	Asthma incidence and	Age; sex; country of birth; place child was born or lived during the first year of life; family history of asthma or

								wheezing	allergies; the number of siblings; child attended preschool or daycare; access to a regular physician or clinic; the child had an ear infection during the first year of life; the child had a viral infection during the first year of life; child took antibiotics during the first year of life; child ever breastfed; current exposure to cats and/or dogs; smoker present in the home at the time of child's birth; current smoker present in the home; proximity to traffic, population density, neighborhood deprivation, percentage of residents who identify as Mexican, and number of total crimes reported in 2004
Fan et al., 2020	China	Cross-sectional	2014-2015	66752	40y+	100/300/500/1000/2000/3000	Per IQR	COPD prevalence	Age, sex, marital status, education background, smoking status, history of secondhand smoking exposure, residence, height, history of tuberculosis, hospital admission due to severe pulmonary disease in childhood, indoor exposure to biomass or coal, workplace exposure to airborne dust or hazardous chemical gases, relative humidity, temperature, and PM2.5 concentrations.
Fuertes et al., 2014	Northern and southern Germany	Cohort	——	5803	0-10y	500	Per IQR	AR incidence and eye and nose symptoms	Age, sex, parental history of atopy, older siblings, maternal smoking during pregnancy, tobacco smoke exposure in the home (1–4 years), parental education, cohort, and geographical area (models for the total population only).
Gernes et al., 2019	Ohio and Kentucky	Cohort	2001-2010	478	0-7y	400	Per IQR	AR incidence	Race, gender, environmental tobacco smoke exposure, traffic-related air pollution exposure, maternal education (7 years), and neighborhood SES (7 years).
Hartley et	Cincinnati	Cohort	2001.10-200	762	0-7y	200/400/800	Per 0.1	Asthma	ECAT, household income, and community deprivation index

al., 2022			3.07					incidence	
Hu et al., 2023	Shanghai	Cross-sectional	2019.04-2019.06	16605	3-12y	250/500/1000	Per IQR	Asthma incidence	Child's age, sex, gestational week, delivery mode, duration of exclusive breastfeeding, miscarriage, SES, residential areas, having plants, passive smoking, child's physical activity, sleeping time, screen exposure time, temperature and PM1
Kuiper et al.,2021	Norway and Sweden	Case-control	2013-2015	3428	18-40y	100/500/1000	Per 0.1	Asthma and rhinitis incidence	O_3, NO_2, age, gender, parental education, and parental asthma
Li et al., 2019	Suzhou, China	Cross- sectional	2014-2015	5643	Middle school students	100/200/500/1000	Per IQR	Current asthma and ever Rhinitis incidence	Child's age and sex, ETS at home, parental education, and parental history of asthma.
Lin et al., 2022	Guangzhou, China	Cohort	2017-2018	522	0-2y	250/500	Per 0.1	AR incidence	Maternal age, maternal employment status, maternal education level, monthly household income, child's sex, birth season, physical activity in pregnancy, and PM2.5 during pregnancy.
Sarkar et al., 2019	England, Wales and Scotland	Cross- sectional	2006.04.04-2010.10.01	96779	39y+	500	Per IQR	COPD prevalence	Demographic characteristics (age, sex, household income, highest qualification, employment status), lifestyle factors (alcohol intake frequency, smoking, residential tenures), neighborhood socioeconomic status (Townsend index), anthropometrics (standing height, body-mass index status), comorbidities (cardiovascular problems, diabetes, parental COPD), and hematological biomarkers (white blood cell counts, neutrophil-to-lymphocyte ratio, and eosinophil-to-basophil ratio)
Sbihi et al., 2015	British Columbia, Canada	Cohort	1999-2012	65000	0-10y	100	Per IQR	Asthma incidence	Maternal age at delivery, birth weight, gestational age, neighborhood-level family income, neighborhood-level

									maternal education, parity, and breastfeeding
Shao et al., 2019	Jiading District, Shanghai	Case-Control	2014-2016	2415	Tumor patients	——	Per 0.1	Lung cancer incidence	Sex, age, smoking and NDVI values
Yang et al., 2023	Taiyuan, Shanxi	Cross- sectional	——	2920	Pre-school children	300/500/1000/3000	Per 0.1	Rhinitis incidence	Age, sex, delivery method, breastfeeding, maternal education, maternal smoking during pregnancy, visible damp, household income, ETS at home, new furniture, hold pet/plant, maternal allergy history, PM2.5, NO_2, O_3, and distance to the trunk road
Xiao et al., 2022	China	Cross-sectional	2012.06-2015.05	50991	20-89y	250/500/1000/1250	Per IQR	COPD incidence	Age, sex, residential area, geographic region, education level, body mass index, smoking history, smoking exposure, the number of smokers living in the home, the season for lung function test, history of tuberculosis, history of pneumonia or bronchitis during childhood, chronic cough during childhood, parental history of respiratory diseases, biomass use, and annual average concentrations of fine particulate matter and ozone.
Zeng et al., 2020	Seven northeastern cities in China	Cross-sectional	2012-2013	59754	Children：10.3±3.6	30/100/300/500/1000	Per 0.1	Asthma incidence and wheezing	Age, gender, parental education, family income, breastfeeding, low birthweight, preterm, residential area, SHS, mold in the home, home coal usage, and family history of asthma.
**Risk Ratios (RR)**									
Lee et al., 2020(a)	Taiwan	Ecological	2006-2014	1173773	General population	250	Per 0.1	Lung cancer mortality	Age, sex ratio, taxable income, precipitation, time trend, and temperature
Lee et al., 2020(b)	Taiwan	Cohort	2003-2011	11281	4-12y	250	Per 0.1	AR incidence	Air temperature, relative humidity, PM2.5 concentrations, socioeconomic status (income tax level as a proxy), road

									network, industrial area, population size, sex ratio, year, season, township urbanization level, and spatial-temporal autocorrelation
Kasdagli et al., 2022	Greece	Ecological	2011	1035 municipal units	General population	1000	Per IQR	COPD mortality	Lung cancer rates, population born in Greece, unemployment rate, population aged 25–64 with upper secondary or tertiary education attainment and urbanicity
Sun et al., 2021	Shanghai	Ecological	2009-2013	841 neighborhoods	General population	1000	Per IQR	lung cancer incidence	Urban form, road traffic, demographic factors, SES factors
Xu et al., 2017	Hong Kong	Ecological	2006-2011	199 (Tertiary Planning Units)	20y+	——	Per IQR	CRD and lung cancer mortality	Age, gender, population density, and area-level socio-economic variables
**Hazard Ratios (HR)**									
Bereziartua et al., 2022	Europe	Cohort	1985-2015	327388	The ELAPSE of 14 sub-cohorts	300/1000	Per 0.1	Lung cancer, and COPD mortality	Age (specified as the underlying time scale), sub-cohort (included as strata), sex (included as strata), calendar year of enrolment (adjusted to account for time-trends in exposure and outcome), individual-level variables: marital status (married/cohabiting, divorced, single, widowed), smoking status (never, former, current), smoking duration (years of smoking) for current smokers, smoking intensity (cigarettes/day) for current smokers, squared smoking intensity, and employment status (yes vs. no) and neighborhood-level SES
Coleman et al., 2021	U.S.A.	Cohort	2000-2016	5529005	18-85y	——	Per IQR	COPD mortality	Median income, median home value, median rent, percent impoverished, percent uninsured, percent unemployed,

									percent less than high school grad, percent high school graduate, percent more than high school graduate, percent rural, percent working class, percent physically active, percent smokers, percent obese, and percent consumers of alcohol
Huang et al., 2022	Taiwan	Cohort	2000-2015	407415	General population	500	Per 0.1	Lung cancer incidence	Age, sex, region, education, history of asthma, history of gout, cigarette smoking, UA, ALP, and AFP
Kayyal-Tarabeia et al., 2022	Israeli	Cohort	1995-2015	144427	General population	500	Per IQR	Lung cancer incidence	Baseline income (reported as continuous and categorized to tertiles), baseline education (personal education variable was classified according to the country-specific coding scheme provided by ISCED-2011 (Unesco Institute for Statistics, 2011) into four categories and in addition a category for children under 15 years old: ISCED 6–8: bachelor to doctoral or equivalent level; ISCED 5: a short cycle of tertiary education; ISCED 1–4: lower secondary to postsecondary non-tertiary education; ISCED 0: pre-primary education or yeshiva)
Klompmaker et al., 2020	Netherlands	Cohort	2013-2017	339633	30y+	300/1000	Per IQR	Lung cancer mortality	Marital status, region of origin, education, paid occupation, standardized household income, physical activity, BMI, smoking status, cigarettes smoked, alcohol status, glasses alcohol, PC4 composite SES, mean income neighborhood, unemployment neighborhood, percentage of immigrants neighborhood, mean income region, unemployment region and percentage of immigrants region
Klompmake	Netherlands	Cohort	2013-2018	10481566	30y+	300/1000	Per IQR	Lung cancer	Marital status, region of origin, standardized household

r et al., 2021								mortality	income, PC4 composite SES, mean income neighborhood, unemployment neighborhood, percentage of immigrants neighborhood, mean income region, unemployment region, and percentage of immigrants region
Rodriguez-Loureiro et al., 2022	Belgian	Cohort	2001-2014	2441566	30y+	300/500/1000	Per IQR	Lung cancer mortality	The baseline hazard and two frailty terms, for the urban areas and for the census tracts within the urban areas, migrant background, educational level, housing tenure, household living arrangement, and area-level socioeconomic position (SEP)
Sakhvidi et al., 2021	French	Cohort	1989-2006	19408	35-50y	100/300/500/1000	Per IQR	Lung cancer incidence	Smoking status, smoking intensity (pack per year), passive smoking, alcohol drinking, socio-occupational status, marital status, body mass index, vegetable consumption, education, occupational exposure to carcinogens, age at enrolment, 10 years cumulative exposure to air pollution (PM2.5), distance to major roads, population density, and deprivation (based on French deprivation index at 2009)
Wang et al., 2022	China	Longitudinal	2011-2018	17574	45y+	1000	Per 0.1	Asthma incidence	Age, sex, region of residence (urban/rural), GDP, BMI, education level, household income, smoking status, alcohol consumption, cooking fuel, disability, sleep duration, time-varying PM2.5, time-varying annual precipitation, O_3, and NO_2
Yu et al., 2023	UK	Cohort	2012-2021	363212	40-70y	500/1000	Per IQR	COPD incidence	Age, sex, body mass index, highest qualification, household income, economic status, smoking status, passive smoking exposure at home, residence, Townsend deprivation index

AR=Allergic rhinitis, COPD= Chronic obstructive pulmonary disease, RD=respiratory disease, CRD=Chronic respiratory disease

Table S4 Risk of bias assessment (WHO 2012*and van Kempen et al., 2018).**

	Bias due to exposure assessment	Bias due to confounding	Bias due to selection of participants	Bias due to health out come assessment	Bias due to not blinded outcome assessment	Total risk of bias
Low	A clear description of the exposure assessment and exposure unit; based on measurements or modeling.	All important confounders are taken into account either through matching or, restriction or in the analysis. (e.g., age, gender, etc.)	Participants randomly sampled from a know n population, AND re sponse rate higher tha n 60%, AND attrition rate less than 20% i n follow-up studies.	The health outcome of interest is objectively measured OR taken from medical records OR taken from a questionnaire or interview using a known scale or validated assessment method.	The health outcome of interest is assessed blind for exposure information in cohort and cross-sectional studies or exposure is assessed blindly for being a case in case-control studies	At least 4 at low risk of bias. One “high” or “unclear” out of five is allowed.
High	Not clear description of the exposure assessment or exposure unit OR/AND performed by unqualified staff	Only 1 or no confounder is taken into account, OR subjects in exposed and unexposed groups differ for one or more important confounders and there is no adjustment in the analysis	No random sampling OR response rate less than 60% OR attrition rate higher than 20%.	The health outcome of interest is self-reported and not assessed using a known scale or validated assessment method	The health outcome and/or exposure assessment is not blinded.	Any other.
Unclear	If not enough	Less than all to > 1	No information to	Not sufficient	Not sufficient	

	information is available to judge the above	important confounders taken into account, OR Insufficient information to decide on one of the above.	judge the above.	information reported to assess the above.	information reported to assess the above.	
Not Apply		NA	NA		NA	

*World Health Organization, WHO Handbook for guideline development. 2012, Geneva: World Health Organization.
**van Kempen, E.; Casas, M.; Pershagen, G.; Foraster, M. WHO Environmental Noise Guidelines for the European Region: A Systematic Review on Environmental Noise and Cardiovascular and Metabolic Effects: A Summary. Int. J. Environ. Res. Public Health 2018, 15, 379.

Table S5 Risk of bias assessment of included studies.

Study	Exposure assessment	Confounding	Selection of participants	Health outcome assessment	Not blinded outcome assessment	Total risk of bias	Event
Andrusaityte et al., 2016	Low	Low	Low	Low	Low	Low	Asthma incidence
Dadvand et al., 2014	Low	Low	High	Low	Low	Low	Asthma and rhinitis incidence
Donovan et al., 2018	Low	Low	Low	Low	Low	Low	Asthma incidence
Dzhambov et al., 2021	Low	Low	Low	Low	Low	Low	Asthma and AR incidence
Eldeirawi et al., 2019	Low	Low	Low	Low	Low	Low	Asthma incidence
Wang et al., 2022	Low	Low	High	Low	Low	Low	Asthma incidence
Hartley et al., 2022	Low	Low	Low	Low	Low	Low	Asthma incidence
Sbihi et al., 2015	Low	Low	High	Low	Low	Low	Asthma incidence
Kuiper et al., 2021	Low	Low	High	High	Low	High	Asthma and rhinitis incidence

Li et al., 2019	Low	Low	Low	Low	Low	Low	Asthma and rhinitis incidence
Rufo et al., 2021	Low	Low	High	Low	Low	Low	Asthma and allergy incidence
Sarkar et al., 2019	Low	Low	Low	Low	Low	Low	COPD incidence
Xiao et al., 2022	Low	Low	Low	Low	Low	Low	COPD incidence
Zeng et al., 2020	Low	Low	Low	Low	Low	Low	Asthma incidence
Hu et al., 2023	Low	Low	Low	High	Low	Low	Asthma incidence
Fuertes et al., 2014	Low	Low	High	High	Low	High	AR incidence
Gernes et al., 2019	Low	Low	High	Low	Low	Low	AR incidence
Lin et al., 2022	Low	Low	High	Low	Low	Low	AR incidence
Lee et al., 2020(b)	Low	Low	Low	Low	Low	Low	AR incidence
Yang et al., 2023	Low	Low	Low	Low	Low	Low	Rhinitis incidence
Yu et al., 2023	Low	Low	Low	Low	Low	Low	COPD incidence
Fan et al., 2020	Low	Low	Low	Low	Low	Low	COPD incidence
Sun et al., 2021	Low	Low	High	Low	Low	Low	Lung cancer incidence
Huang et al., 2022	Low	Low	Low	Low	Low	Low	Lung cancer incidence
Kayyal-Tarabeia et al., 2022	Low	Low	Low	Low	Low	Low	Lung cancer incidence
Sakhvidi et al., 2021	Low	Low	Low	Low	Low	Low	Lung cancer incidence
Bereziartua et al., 2022	Low	Low	Low	Low	Low	Low	Lung cancer, and COPD mortality
Coleman et al., 2021	Low	Low	Low	Low	Low	Low	COPD mortality
Kasdagli et al., 2022	Low	Low	Low	Low	Low	Low	COPD mortality
Xu et al., 2017	Low	Low	Low	Low	Low	Low	CRD and lung cancer mortality
Lee et al., 2020(a)	Low	Low	Low	Low	Low	Low	Lung cancer mortality

Klompmakeret al., 2020	Low	Low	Low	Low	Low	Low	Lung cancer mortality
Klompmakeret al., 2021	Low	Low	Low	Low	Low	Low	Lung cancer mortality
Rodriguez-Loureiro et al., 2022	Low	Low	Low	Low	Low	Low	Lung cancer mortality
Shao et al., 2019	Low	Low	Low	Low	Low	Low	Lung cancer prevalence

Table S6. GRADE assessment of the quality of evidence reported the associations between greenness exposure and CRDs outcomes.

Outcome	Number of studies	Design	Design Risk of bias	Inconsistency	Indirectness	Imprecision	Other considerations	Number of participants	RR(95%CI)	Quality
Asthma incidence	9	Observational studies	Not serious	$I^2>50\%$	Not serious	Not serious	None	172206	0.92 (0.85, 0.98)	⊕⊕OO LOW
Asthma prevalence	4	Observational studies	Not serious	$I^2>50\%$	Not serious	Not serious	None	55435	0.89 (0.74, 1.08)	⊕⊕⊕O VERY LOW
AR incidence	6	Observational studies	Not serious	$I^2>50\%$	Not serious	Not serious	None	27155	1.02 (0.97, 1.08)	⊕OOO VERY LOW
AR prevalence	2	Observational studies	Not serious	$I^2>50\%$	Not serious	Not serious	None	4429	0.91 (0.64, 1.29)	⊕OOO VERY LOW
COPD incidence	2	Observational studies	Not serious	$I^2>90\%$	Not serious	Not serious	None	414203	0.92 (0.83, 1.03)	⊕OOO VERY LOW
COPD prevalence	2	Observational studies	Not serious	$I^2>90\%$	Not serious	Not serious	None	163531	1.00 (0.90, 1.12)	⊕OOO VERY LOW

Lung cancer incidence	5	Observational studies	Not serious	I²>90%	Not serious	Not serious	None	3,519,565	0.62 (0.40, 0.95)	⊕⊕OO LOW
COPD mortality	3	Observational studies	Not serious	Not serious	Not serious	Not serious	None	7,190,543	0.95 (0.92, 0.99)	⊕⊕⊕O MODERATE
Lung cancer mortality	6	Observational studies	Not serious	I² >50%	Not serious	Not serious	None	21,621,026	0.98 (0.96, 1.01)	⊕OOO VERY LOW

Abbreviations: CRD: Chronic respiratory disease; AR: Allergic rhinitis; COPD: Chronic obstructive pulmonary disease.

Table S7. GRADE assessment of the quality of age subgroups.

Outcome	Number of studies	Design	Design Risk of bias	Inconsistency	Indirectness	Imprecision	Other considerations	Number of participants	RR (95%CI)	Quality
**Aged 0-7y**										
Asthma incidence	4	Observational studies	Not serious	I² >50%	Not serious	Not serious	None	68,301	0.98 (0.80, 1.20)	⊕OOO VERY LOW
AR incidence	2	Observational studies	Not serious	Not serious	Not serious	Not serious	None	1,000	0.99 (0.83, 1.19)	⊕⊕OO LOW
**Aged 7-12y**										
Asthma incidence	2	Observational studies	Not serious	I² >50%	Not serious	Not serious	None	4,429	0.91 (0.63, 1.30)	⊕OOO VERY LOW
AR incidence	2	Observational studies	Not serious	I² >50%	Not serious	Not serious	None	4,429	0.91 (0.64, 1.29)	⊕OOO VERY

										LOW
**Aged 12-18y**										
Asthma incidence	2	Observational studies	Not serious	Not serious	Not serious	Not serious	None	65,397	0.91 (0.83, 0.99)	⊕⊕⊕O MODERATE
**Aged 40-65y**										
COPD incidence/ prevalence	3	Observational studies	Not serious	I^2>90%	Not serious	Not serious	None	526,743	0.97 (0.91, 1.05)	⊕OOO VERY LOW
**Aged 65y+**										
COPD incidence/ prevalence	3	Observational studies	Not serious	I^2>50%	Not serious	Not serious	None	480,955	0.91 (0.79, 1.04)	⊕OOO VERY LOW

Abbreviations: AR: Allergic rhinitis; COPD: Chronic obstructive pulmonary disease.

Table S8. GRADE assessment of the quality of buffer subgroups.

Outcome	Number of studies	Design	Design Risk of bias	Inconsistency	Indirectness	Imprecision	Other considerations	Number of participants	RR(95%CI)	Quality
**NDVI 200-300m**										
Asthma incidence	9	Observational studies	Not serious	I^2>50%	Not serious	Not serious	None	142,730	0.92 (0.86, 0.98)	⊕⊕OO LOW
AR incidence	5	Observational studies	Not serious	I^2>50%	Not serious	Not serious	None	24,052	1.02 (0.95, 1.09)	⊕OOO VERY LOW
Lung cancer	5	Observational studies	Not serious	I^2>50%	Not serious	Not serious	None	14,763,926	0.98 (0.94, 1.01)	⊕OOO VERY

mortality										LOW
**NDVI 400-500m**										
Asthma incidence	9	Observational studies	Not serious	I^2 >50%	Not serious	Not serious	None	94,025	0.93 (0.85, 1.01)	⊕OOO VERY LOW
AR incidence	7	Observational studies	Not serious	Not serious	Not serious	Not serious	None	20,303	0.99 (0.94, 1.03)	⊕⊕OO LOW
COPD incidence	4	Observational studies	Not serious	I^2 >50%	Not serious	Not serious	None	577,734	0.95 (0.89, 1.02)	⊕OOO VERY LOW
Lung cancer incidence/ prevalence	3	Observational studies	Not serious	I^2 >90%	Not serious	Not serious	None	571,250	0.70 (0.46, 1.06)	⊕OOO VERY LOW
**NDVI 800-1000m**										
Asthma incidence	8	Observational studies	Not serious	I^2 >50%	Not serious	Not serious	None	108,195	0.87 (0.81, 0.93)	⊕⊕OO LOW
AR incidence	4	Observational studies	Not serious	Not serious	Not serious	Not serious	None	21,875	0.99 (0.91, 1.06)	⊕⊕OO LOW
COPD incidence	3	Observational studies	Not serious	I^2 >90%	Not serious	Not serious	None	480,955	0.92 (0.83, 1.03)	⊕OOO VERY LOW
Lung cancer incidence/	2	Observational studies	Serious	I^2 >90%	Not serious	Not serious	None	3,479,408	0.20 (0.01, 4.48)	⊕OOO VERY LOW

prevalence										
Lung cancer mortality	4	Observational studies	Not serious	I^2 >50%	Not serious	Not serious	None	15,590,153	0.98 (0.84, 1.03)	⊕OOO VERY LOW
COPD mortality	2	Observational studies	Not serious	Not serious	Not serious	Not serious	None	1,661,538	0.93 (0.88, 0.98)	⊕⊕⊕O MODERATE

Abbreviations: NDVI: Normalized difference vegetation index; AR: Allergic rhinitis; COPD: Chronic obstructive pulmonary disease.

Table S9. Sensitivity analysis of the meta-analytic association between NDVI and CRDs incidence/prevalence.

**Excluded study**	**Effect estimates(95%CI)**
**Asthma incidence**	
Andrusaityte et al., 2016	0.90 (0.84,0.97)
Eldeirawi el a., 2019	0.90 (0.84,0.97)
Hartley, K., et al., 2022	0.91 (0.85,0.98)
Hu et al., 2023	0.92 (0.84,1.00)
Kuiper et al., 2021	0.91 (0.84,0.99)
Li et al., 2019	0.91 (0.85,0.98)
Sbihi et al., 2015	0.91 (0.84,0.99)
Wang et al., 2022	0.92 (0.85,1.00)
Zeng et al., 2020(August)	0.93 (0.87,1.00)
Zeng et al., 2020(October)	0.93 (0.87,1.00)
**Asthma prevalence**	

Cavaleiro Rufo et al., 2020	0.93 (0.85,1.05)
Dadvand et al., 2014	0.78 (0.56,1.08)
Donovan et al., 2018	0.78 (0.50,1.20)
Dzhambov et al., 2021	0.93 (0.75,1.15)
**COPD incidence**	
Xiao et al., 2022	0.87 (0.82,0.92)
Yu,et al., 2022	0.97 (0.95,0.99)
**COPD prevalence**	
Fan et al., 2020	1.06 (1.02,1.10)
Sarkar et al., 2019	0.95 (0.93,0.97)
**AR incidence**	
Fuertes et al., 2014	1.02 (0.95,1.10)
Gernes et al., 2019	1.03 (0.97,1.09)
Kuiper et al., 2021	1.04 (0.98,1.10)
Lee et al., 2020(b)	1.00 (0.96,1.04)
Li et al., 2019	1.03 (0.97,1.09)
Lin et al., 2022	1.02 (0.96,1.08)
**AR prevalence**	
Dadvand et al., 2014	0.73 (0.51,1.04)
Dzhambov et al., 2021	1.05 (0.93,1.19)

**Lung cancer incidence**	
Huang et al., 2022	0.50 (0.27,0.93)
Kayyal-Tarabeia et al., 2022	0.87 (0.69,1.10)
Sakhvidi et al., 2021	0.52 (0.29,0.94)
Shao et al., 2019	0.51 (0.29,0.91)
Sun et al., 2021	0.75 (0.49,1.14)

Table S10. Sensitivity analysis of the meta-analytic association between NDVI and CRDs mortality.

Excluded study	Effect estimates(95%CI)
**COPD mortality**	
Bereziartua et al., 2022	0.95 (0.90,1.01)
Coleman et al., 2021	0.93 (0.88,0.98)
Kasdagli et al., 2022	0.97 (0.93,1.01)
**Lung cancer mortality**	
Bereziartua et al., 2022	0.99 (0.96,1.02)
Klompmaker et al., 2020	0.98 (0.95,1.01)
Klompmaker et al., 2021	0.99 (0.97,1.02)
Lee et al., 2020	0.99 (0.96,1.01)
Rodriguez-Loureiro et al., 2022	0.98 (0.94,1.02)

Xu et al., 2017	0.98 (0.95,1.00)

Table S11. Sensitivity analysis of the age subgroups meta-analytic association between NDVI and CRDs.

Types of CRDs	Excluded study	Effect estimates(95%CI)
**0-7years old**		
Asthma incidence/prevalence	Andrusaityte et al., 2016	0.90 (0.67,1.21)
	Cavaleiro Rufo et al., 2020	0.96 (0.93,1.00)
	Hartley et al., 2022	0.95 (0.71,1.27)
	Sbihi et al., 2015	0.92 (0.60,1.43)
AR incidence	Gernes et al., 2019	1.08 (0.79,1.48)
	Lin et al., 2022	0.95 (0.76,1.19)
**8-12 years old**		
Asthma prevalence	Dadvand et al., 2014	1.06 (0.84,1.34)
	Dzhambov et al., 2021	0.73 (0.50,1.07)
AR prevalence	Dadvand et al., 2014	1.05 (0.93,1.19)
	Dzhambov et al., 2021	0.73 (0.51,1.04)
**13-18 years old**		
Asthma incidence	Li et al., 2019	1.04 (0.67,1.61)

	Zeng et al., 2020	0.90 (0.82,0.99)
**40-65 years old**		
COPD incidence/prevalence	Fan et al., 2020	0.95 (0.92,0.97)
	Sarkar et al., 2019	0.98 (0.86,1.12)
	Yu,et al., 2022	1.00 (0.90,1.10)
**Over 65 years old**		
COPD incidence/prevalence	Fan et al., 2020	0.85 (0.77,0.95)
	Xiao et al., 2022	0.90 (0.75,1.09)
	Yu,et al., 2022	0.98 (0.92,1.04)

Table S12. Sensitivity analysis of the buffer subgroups meta-analytic association between NDVI and CRDs.

**Types of CRDs**	**Excluded study**	**Effect estimates(95%CI)**
**NDVI 200-300m**		
Asthma incidence/prevalence	Andrusaityte et al., 2016	0.90 (0.85,0.96)
	Dadvand et al., 2014	0.92 (0.86,0.98)
	Donovan et al., 2018	0.93 (0.85,1.01)
	Eldeirawi el al., 2019	0.92 (0.86,0.98)
	Hartley et al., 2022	0.92 (0.86,0.99)
	Hu, Y., et al., 2023	0.93 (0.86,1.01)
	Kuiper et al., 2021	0.92 (0.85,0.99)

	Li et al., 2019	0.91 (0.85,0.98)
	Zeng et al., 2020(August)	0.94 (0.87,1.02)
	Zeng et al., 2020(October)	0.93 (0.89,0.98)
AR incidence/prevalence	Dadvand et al., 2014	1.02 (0.95,1.10)
	Kuiper et al., 2021	1.02 (0.93,1.11)
	Lee et al., 2020(b)	0.99 (0.94,1.04)
	Li et al., 2019	1.04 (0.97,1.10)
	Lin et al., 2022	1.01 (0.94,1.09)
Lung cancer mortality	Bereziartua et al. 2022	0.98 (0.94,1.01)
	Klompmaker et al., 2020	0.98 (0.94,1.03)
	Klompmaker et al., 2021	0.96 (0.94,0.99)
	Lee et al., 2020	0.97 (0.93,1.00)
	Rodriguez-Loureiro et al., 2022	0.99 (0.95,1.03)
**NDVI 400-500m**		
Asthma incidence/prevalence	Andrusaityte et al., 2016	0.91 (0.83,0.99)
	Dadvand et al., 2014	0.92 (0.84,1.01)
	Donovan et al., 2018	0.94 (0.86,1.03)
	Eldeirawi el al., 2019	0.94 (0.86,1.03)
	Hartley et al., 2022	0.92 (0.84,1.00)
	Hu et al., 2023	0.95 (0.84,1.07)

	Kuiper et al., 2021	0.91 (0.83,0.99)
	Li et al., 2019	0.92 (0.83,1.02)
	Zeng et al., 2020(August)	0.92 (0.84,1.01)
	Zeng et al., 2020(October)	0.96 (0.87,1.06)
AR incidence/prevalence	Dadvand et al., 2014	0.98 (0.93,1.03)
	Dzhambov et al., 2021	0.99 (0.95,1.04)
	Fuertes et al., 2014	0.98 (0.93,1.03)
	Gernes et al., 2019	0.99 (0.94,1.03)
	Kuiper et al., 2021	0.98 (0.91,1.05)
	Li et al., 2019	0.99 (0.94,1.03)
	Lin et al., 2022	0.98 (0.94,1.03)
COPD incidence/prevalence	Fan et al., 2020	0.92 (0.86,0.98)
	Sarkar et al., 2019	0.97 (0.90,1.06)
	Xiao et al., 2022	0.95 (0.85,1.06)
	Yu et al., 2022	0.98 (0.90,1.06)
COPD incidence	Huang et al., 2022	0.60 (0.25,1.44)
	Kayyal-Tarabeia et al., 2022	0.95 (0.91, 0.99)
	Sakhvidi et al., 2021	0.60 (0.25,1.48)
**NDVI 800-1000m**		
Asthma incidence/prevalence	Dadvand et al., 2014	0.88 (0.82,0.95)

	Donovan et al., 2018	0.88 (0.81,0.96)
	Hartley et al., 2022	0.86 (0.80,0.93)
	Hu et al., 2023	0.84 (0.79,0.90)
	Kuiper et al., 2021	0.86 (0.80,0.93)
	Li et al., 2019	0.85 (0.79,0.91)
	Wang et al., 2022	0.87 (0.80,0.94)
	Zeng et al., 2020(August)	0.87 (0.81,0.94)
	Zeng et al., 2020(October)	0.87 (0.81,0.94)
AR incidence/prevalence	Dadvand et al., 2014	0.95 (0.84,1.06)
	Dzhambov et al., 2021	1.00 (0.94,1.05)
	Kuiper et al., 2021	0.95 (0.80,1.12)
	Li et al., 2019	0.99 (0.89,1.09)
COPD incidence/prevalence	Fan et al., 2020	0.92 (0.83,1.03)
	Xiao et al., 2022	0.96 (0.79,1.17)
	Yu et al., 2022	1.01 (0.93,1.10)
Lung cancer incidence	Sakhvidi et al., 2021	0.04 (0.01, 0.16)
	Sun et al., 2021	0.88 (0.74, 1.06)
Lung cancer mortality	Bereziartua et al. 2022	0.99 (0.93,1.05)
	Klompmaker et al., 2020	0.97 (0.93,1.01)
	Klompmaker et al., 2021	0.97 (0.92,1.02)

COPD mortality	Rodriguez-Loureiro et al., 2022	1.00 (0.96,1.04)
	Bereziartua et al. 2022	0.92 (0.86, 0.98)
	Kasdagli et al., 2022	0.94 (0.87, 1.02)

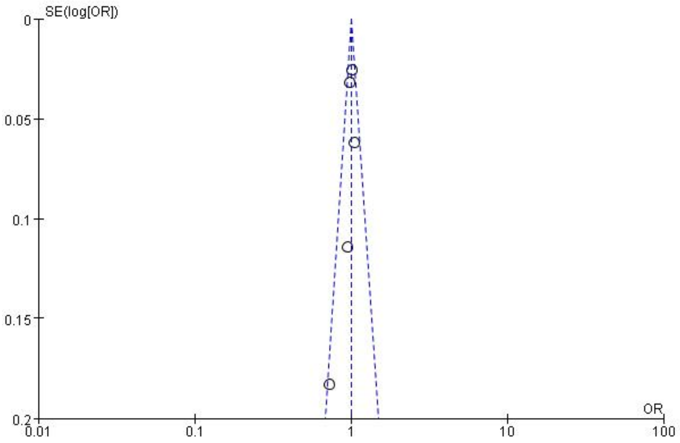

(a)

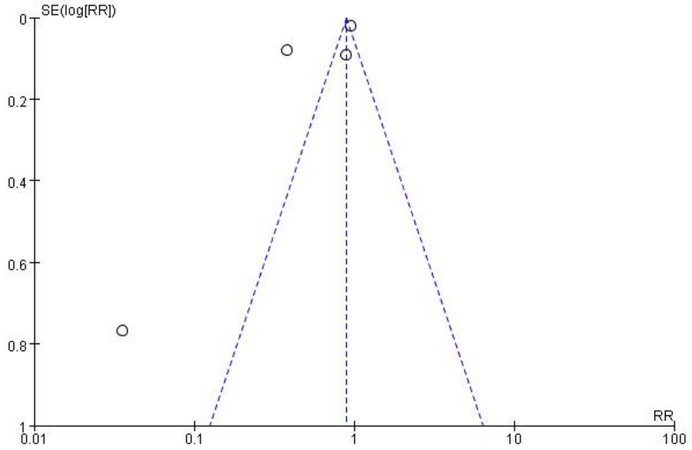

(b)

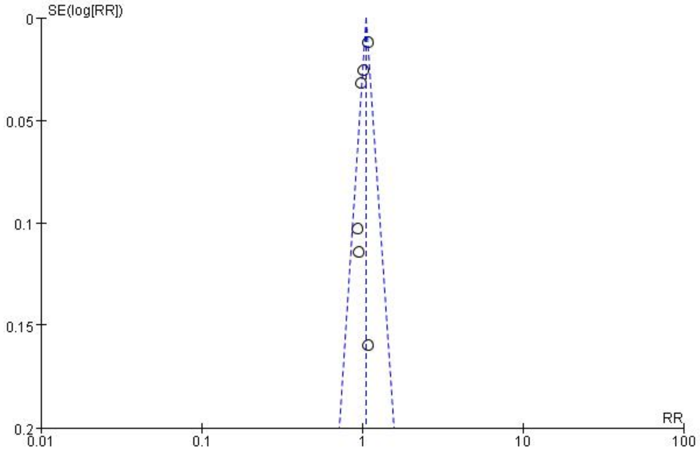

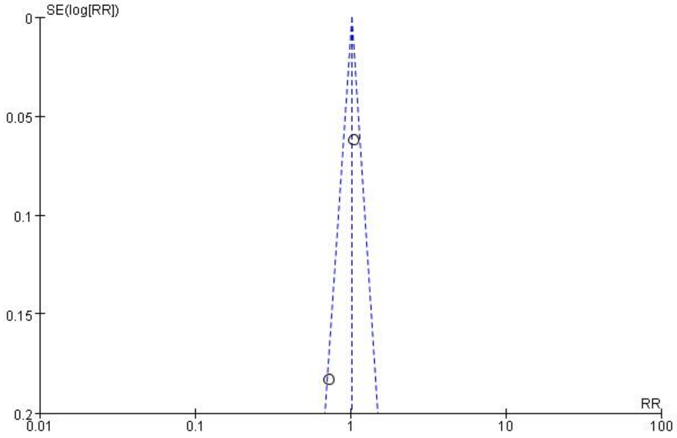

(c) (d)

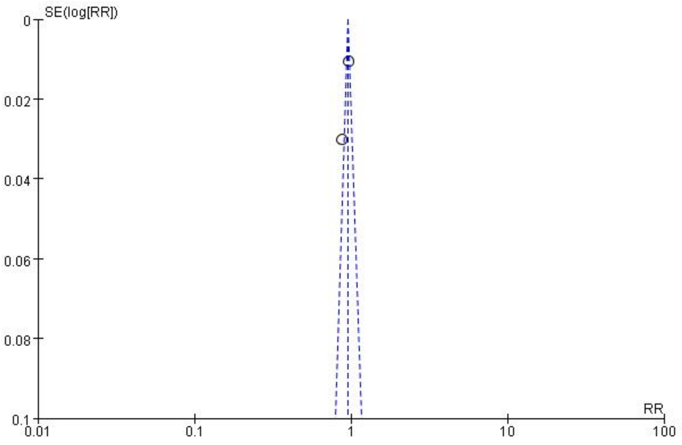
SE(log[RR])
0
0.02
0.04
0.06
0.08
0.1
RR
0.01
0.1
1
10
100

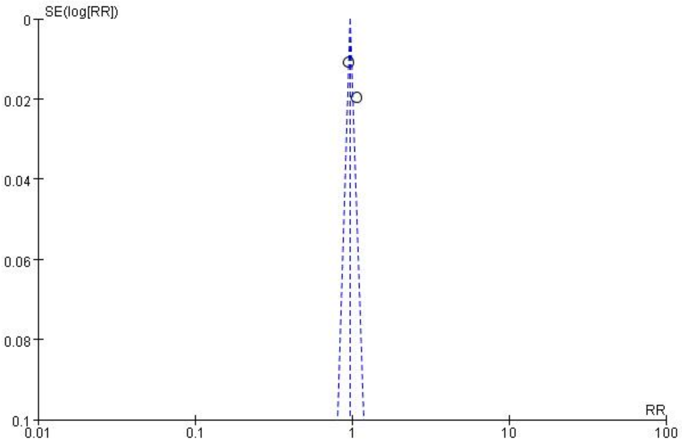
SE(log[RR])
0
0.02
0.04
0.06
0.08
0.1
RR
0.01
0.1
1
10
100

(e) (f)

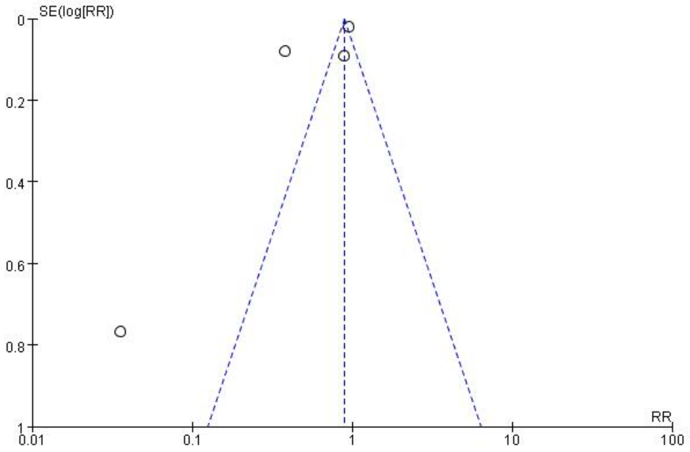
SE(log[RR])
0
0.2
0.4
0.6
0.8
1
RR
0.01
0.1
1
10
100

(g)

Figure S1. Funnel Plots for greenness exposure and CRDs incidence/prevalence. (a) asthma incidence; (b) asthma prevalence; (c) AR incidence; (d) AR prevalence; (e) COPD incidence; (f) COPD prevalence; (g) lung cancer incidence.

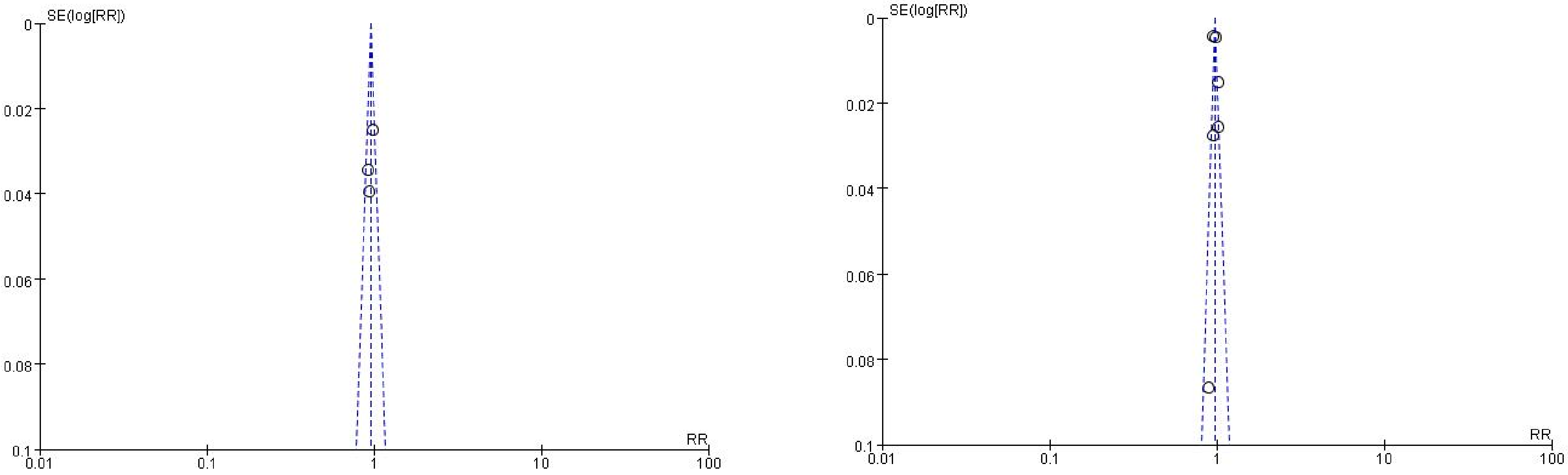

(a) (b)

Figure S2. Funnel Plots for greenness exposure and CRDs mortality. (a) COPD mortality; (b) lung cancer mortality.

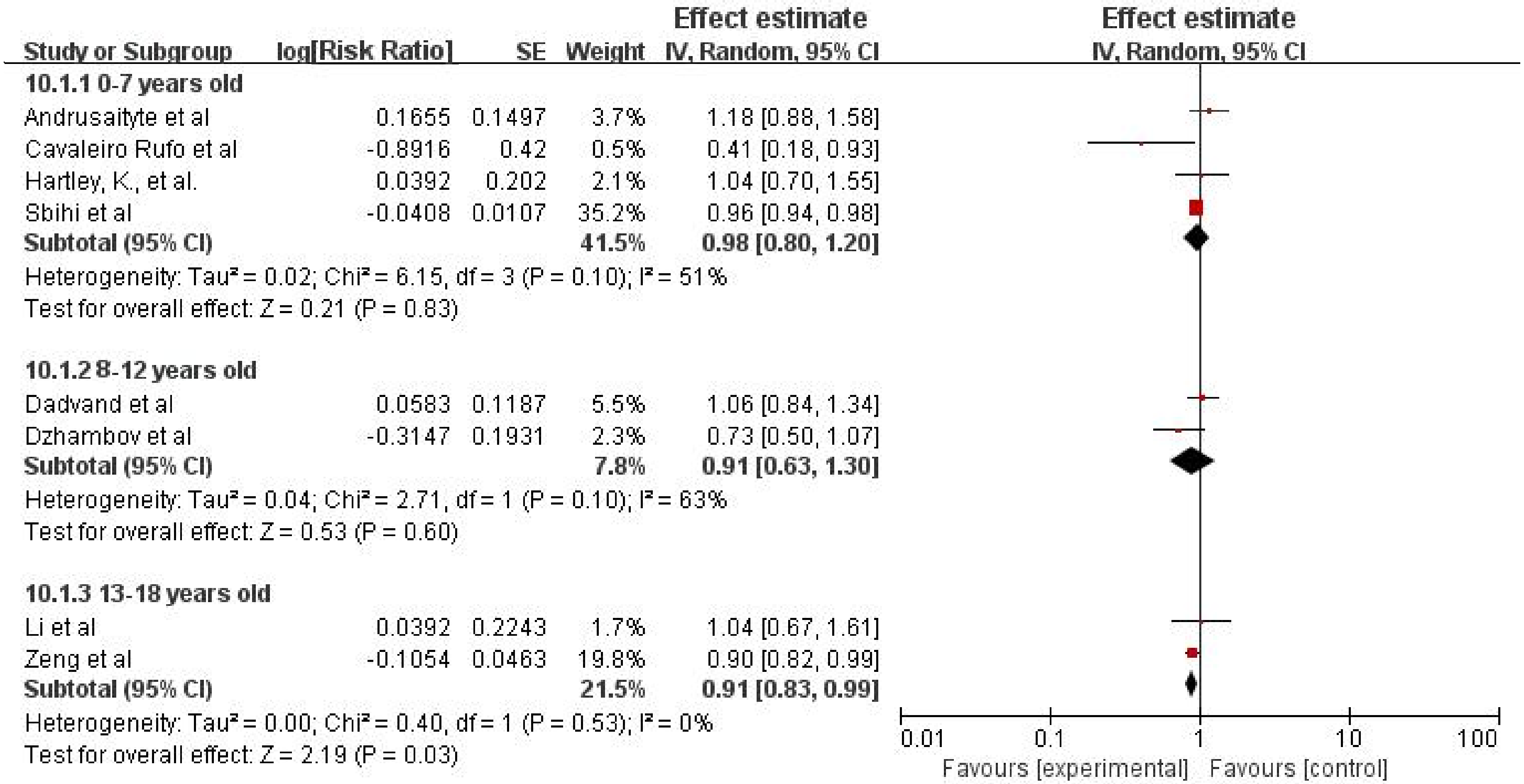

Figure S3. Age subgroup analysis of asthma incidence/prevalence and NDVI for increments of 0.1. NDVI, Normalized Difference Vegetation Index.

Study or Subgroup	log[Risk Ratio]	SE	Weight	Effect estimate IV, Random, 95% CI
**6.2.1 0-7 years old**				
Gernes et al	-0.0513	0.1139	5.2%	0.95 [0.76, 1.19]
Lin, L., et al.	0.077	0.1595	2.6%	1.08 [0.79, 1.48]
**Subtotal (95% CI)**			**7.8%**	**0.99 [0.83, 1.19]**
Heterogeneity: Tau² = 0.00; Chi² = 0.43, df = 1 (P = 0.51); I² = 0%				
Test for overall effect: Z = 0.09 (P = 0.93)				
**6.2.2 8-12 years old**				
Dadvand et al	0.0488	0.0619	17.6%	1.05 [0.93, 1.19]
Dzhambov et al	-0.3147	0.183	2.0%	0.73 [0.51, 1.04]
**Subtotal (95% CI)**			**19.6%**	**0.91 [0.64, 1.29]**
Heterogeneity: Tau² = 0.05; Chi² = 3.54, df = 1 (P = 0.06); I² = 72%				
Test for overall effect: Z = 0.52 (P = 0.60)				

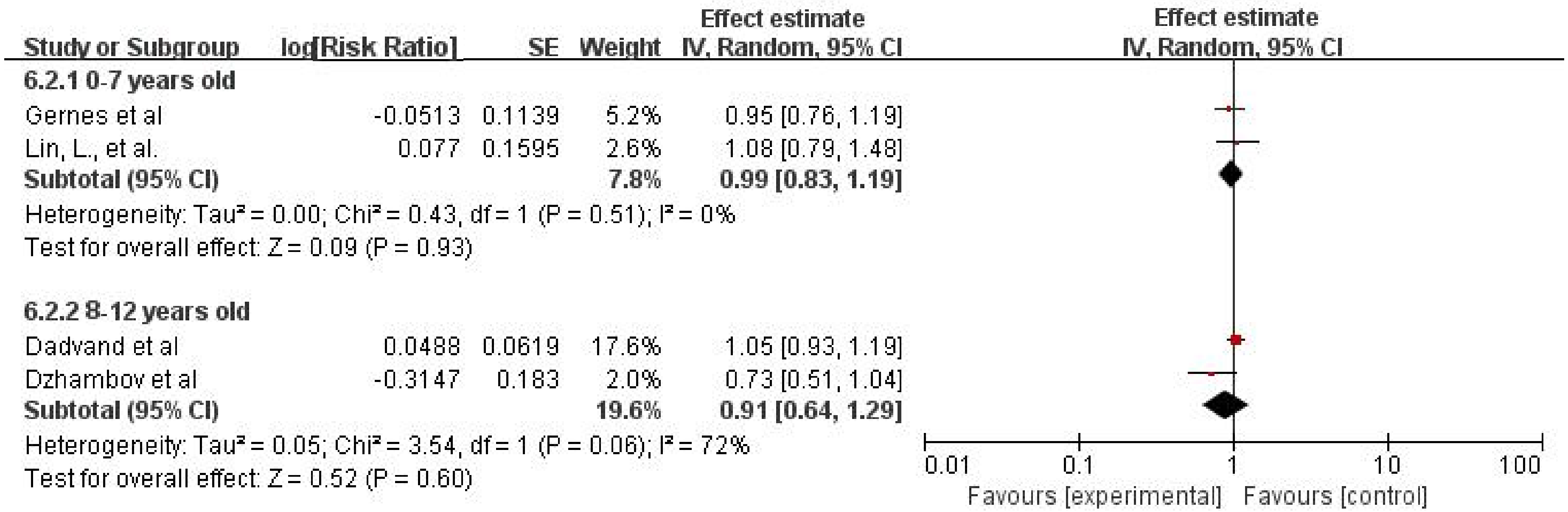

Figure S4. Age subgroup analysis of AR incidence/prevalence and NDVI for increments of 0.1. NDVI, Normalized Difference Vegetation Index; AR, allergic rhinitis.

Study or Subgroup	log[Risk Ratio]	SE	Weight	Effect estimate IV, Random, 95% CI
**8.2.1 40-65 years old**				
Fan et al	0.0488	0.0198	19.5%	1.05 [1.01, 1.09]
Sarkar et al	-0.0513	0.0109	20.7%	0.95 [0.93, 0.97]
Yu,et al.	-0.0834	0.0285	17.9%	0.92 [0.87, 0.97]
**Subtotal (95% CI)**			**58.0%**	**0.97 [0.91, 1.05]**
Heterogeneity: Tau² = 0.00; Chi² = 23.01, df = 2 (P < 0.0001); I² = 91%				
Test for overall effect: Z = 0.74 (P = 0.46)				
**8.2.2 over 65 years old**				
Fan et al	-0.0101	0.0319	17.2%	0.99 [0.93, 1.05]
Xiao et al	-0.0834	0.0777	8.9%	0.92 [0.79, 1.07]
Yu,et al.	-0.1985	0.0388	15.8%	0.82 [0.76, 0.88]
**Subtotal (95% CI)**			**42.0%**	**0.91 [0.79, 1.04]**
Heterogeneity: Tau² = 0.01; Chi² = 14.07, df = 2 (P = 0.0009); I² = 86%				
Test for overall effect: Z = 1.41 (P = 0.16)				

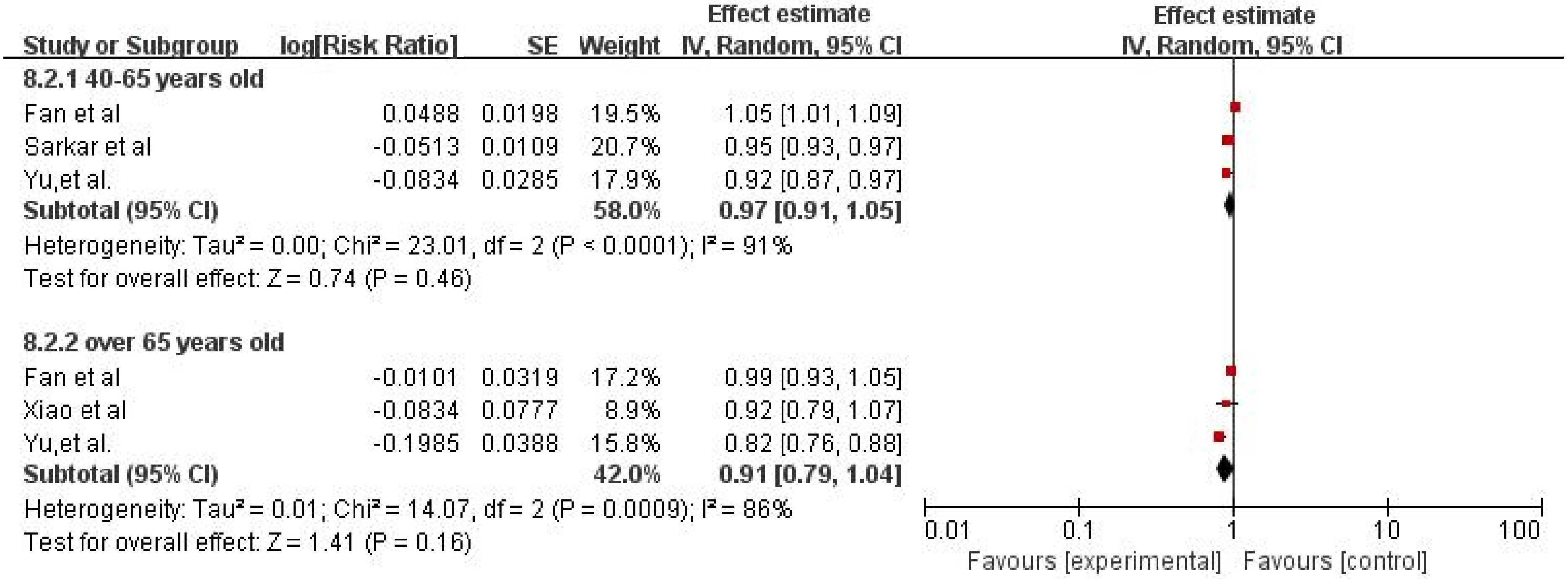

Figure S5. Age subgroup analysis of COPD incidence/prevalence and NDVI for increments of 0.1. NDVI, Normalized Difference Vegetation Index; COPD, Chronic Obstructive Pulmonary Diseases.

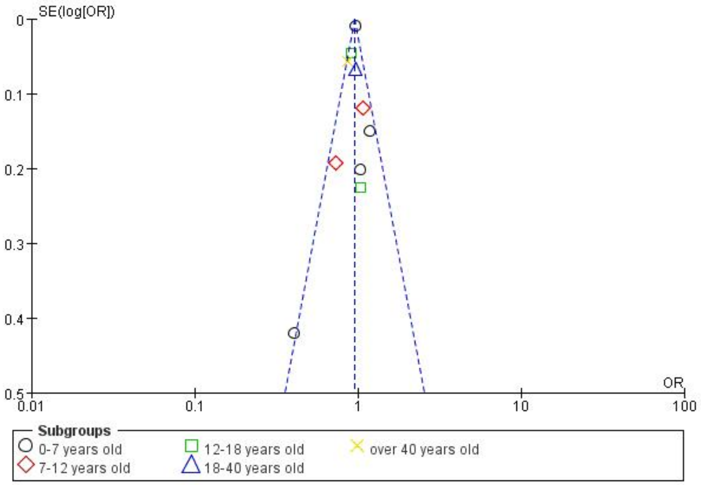

(a)

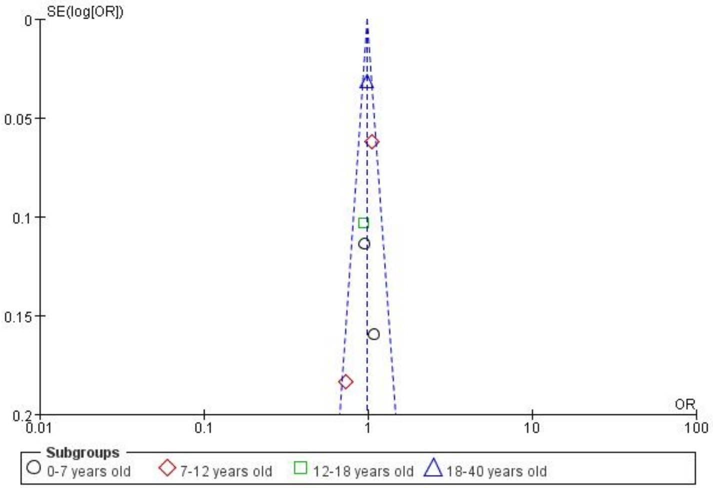

(b)

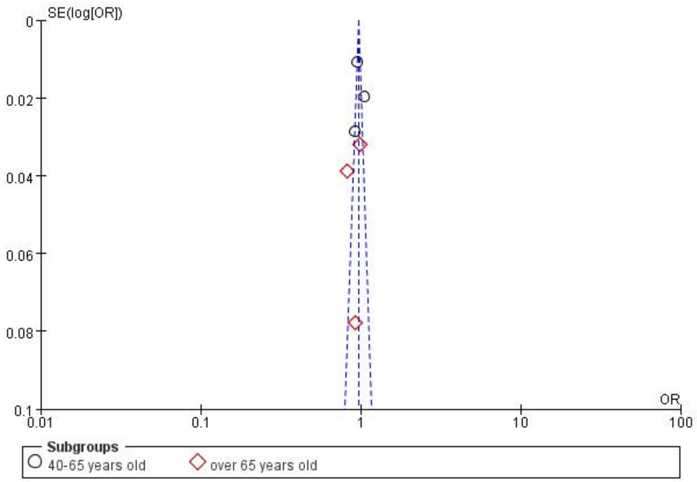

(c)

Figure S6. Funnel Plots for age subgroups. (a) asthma incidence/prevalence; (b) AR in cidence/prevalence; (c) COPD incidence/prevalence.

Study or Subgroup	log[Risk Ratio]	SE	Weight	Effect estimate IV, Random, 95% CI
**10.2.1 300m**				
Andrusaityte et al	0.1906	0.1234	2.1%	1.21 [0.95, 1.54]
Dadvand et al	0	0.1203	2.2%	1.00 [0.79, 1.27]
Donovan et al	-0.0726	0.0224	7.5%	0.93 [0.89, 0.97]
Eldeirawi el al	0.0953	0.1754	1.2%	1.10 [0.78, 1.55]
Hartley, K., et al.	-0.0305	0.1664	1.3%	0.97 [0.70, 1.34]
Hu, Y., et al.	-0.1165	0.0418	6.2%	0.89 [0.82, 0.97]
Kuiper et al	-0.0305	0.0674	4.4%	0.97 [0.85, 1.11]
Li et al	0.0953	0.1376	1.8%	1.10 [0.84, 1.44]
Zeng et al(August)	-0.1393	0.0302	7.0%	0.87 [0.82, 0.92]
Zeng et al(October)	-0.2614	0.0414	6.2%	0.77 [0.71, 0.84]
**Subtotal (95% CI)**			**40.2%**	**0.92 [0.86, 0.98]**
Heterogeneity: Tau² = 0.01; Chi² = 29.06, df = 9 (P = 0.0006); I² = 69%				
Test for overall effect: Z = 2.41 (P = 0.02)				
**10.2.3 500m**				
Andrusaityte et al	0.1655	0.1497	1.6%	1.18 [0.88, 1.58]
Dadvand et al	0.0296	0.1354	1.8%	1.03 [0.79, 1.34]
Dzhambov et al	-0.3711	0.2069	0.9%	0.69 [0.46, 1.04]
Eldeirawi el al	-0.3567	0.2032	0.9%	0.70 [0.47, 1.04]
Hartley, K., et al.	0.1222	0.1762	1.2%	1.13 [0.80, 1.60]
Hu, Y., et al.	-0.1508	0.0433	6.1%	0.86 [0.79, 0.94]
Kuiper et al	0.0953	0.1197	2.2%	1.10 [0.87, 1.39]
Li et al	-0.0305	0.0674	4.4%	0.97 [0.85, 1.11]
Zeng et al(August)	0.01	0.1384	1.8%	1.01 [0.77, 1.32]
Zeng et al(October)	-0.1985	0.0321	6.9%	0.82 [0.77, 0.87]
**Subtotal (95% CI)**			**27.9%**	**0.93 [0.85, 1.01]**
Heterogeneity: Tau² = 0.01; Chi² = 21.37, df = 9 (P = 0.01); I² = 58%				
Test for overall effect: Z = 1.67 (P = 0.09)				
**10.2.4 1000m**				
Dadvand et al	-0.2485	0.048	5.8%	0.78 [0.71, 0.86]
Dzhambov et al	-0.2107	0.0393	6.4%	0.81 [0.75, 0.87]
Hartley, K., et al.	0.0392	0.202	0.9%	1.04 [0.70, 1.55]
Hu, Y., et al.	-0.0619	0.0395	6.4%	0.94 [0.87, 1.02]
Kuiper et al	0.0392	0.2243	0.8%	1.04 [0.67, 1.61]
Li et al	-0.0305	0.0674	4.4%	0.97 [0.85, 1.11]
Wang, J. W., et al.	-0.1508	0.0564	5.2%	0.86 [0.77, 0.96]
Zeng et al(August)	-0.2744	0.1936	1.0%	0.76 [0.52, 1.11]
Zeng et al(October)	-0.3147	0.1931	1.0%	0.73 [0.50, 1.07]
**Subtotal (95% CI)**			**31.9%**	**0.87 [0.81, 0.93]**
Heterogeneity: Tau² = 0.01; Chi² = 17.44, df = 8 (P = 0.03); I² = 54%				
Test for overall effect: Z = 3.97 (P < 0.0001)				

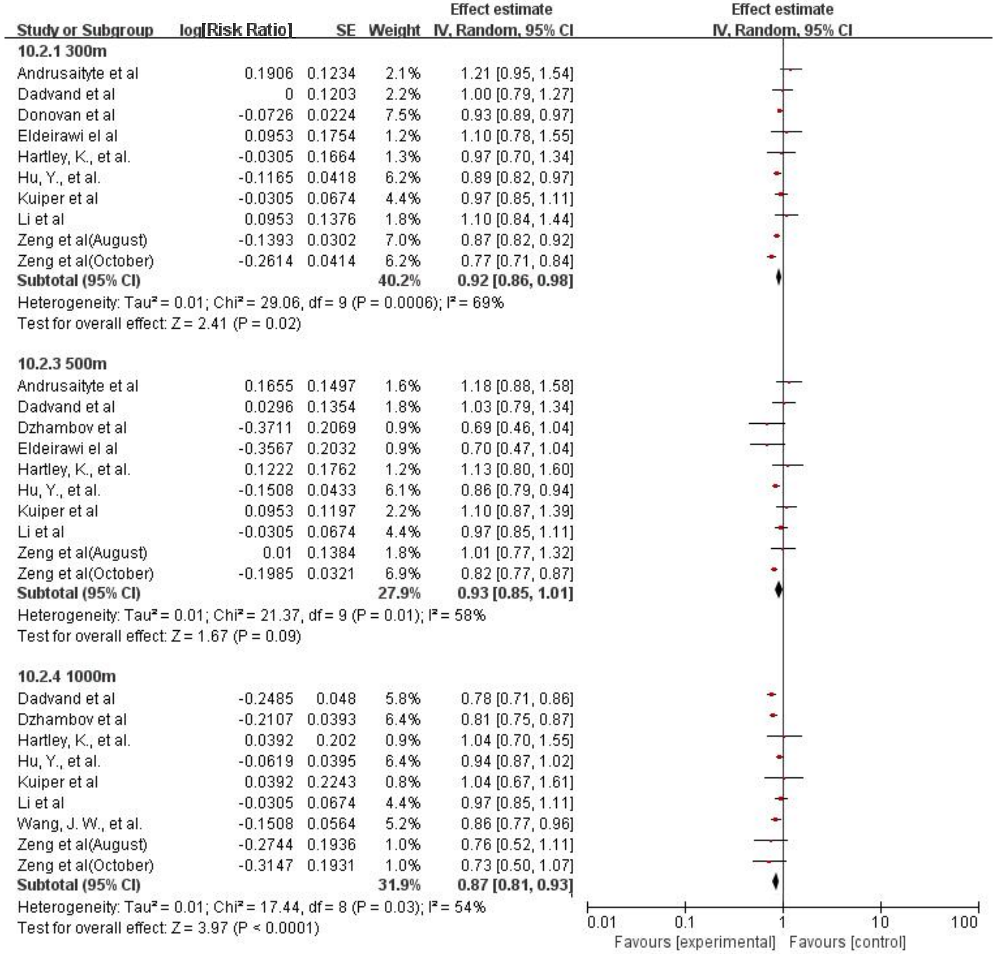

Figure S7. Buffer subgroup analysis of asthma incidence/prevalence and NDVI for increments of 0.1. NDVI, Normalized Difference Vegetation Index.

Study or Subgroup	log[Risk Ratio]	SE	Weight	Effect estimate IV, Random, 95% CI
**6.1.2 300m**				
Dadvand et al(250m rhinoconjunctivitis) 2014	-0.0202	0.0613	6.7%	0.98 [0.87, 1.11]
Kuiper et al (300m rhinitis) 2021	0	0.0316	11.8%	1.00 [0.94, 1.06]
Lee et al (b)	0.0807	0.0119	15.4%	1.08 [1.06, 1.11]
Li et al(200m buffer) 2019	-0.0619	0.0574	7.2%	0.94 [0.84, 1.05]
Lin, L., et al.	0.077	0.2286	0.8%	1.08 [0.69, 1.69]
**Subtotal (95% CI)**			**41.9%**	**1.02 [0.95, 1.09]**
Heterogeneity: Tau² = 0.00; Chi² = 12.77, df = 4 (P = 0.01); I² = 69%				
Test for overall effect: Z = 0.45 (P = 0.65)				
**6.1.3 500m**				
Dadvand et al(500m rhinoconjunctivitis) 2014	0.0198	0.0582	7.1%	1.02 [0.91, 1.14]
Dzhambov et al(500m allergic rhinitis) 2021	-0.1863	0.1093	3.0%	0.83 [0.67, 1.03]
Fuertes et al (allergic rhinitis) 2014	0.0296	0.0745	5.3%	1.03 [0.89, 1.19]
Gernes et al(400m buffer) 2019	-0.0513	0.1139	2.8%	0.95 [0.76, 1.19]
Kuiper et al (500m rhinitis) 2021	-0.0101	0.0319	11.7%	0.99 [0.93, 1.05]
Li et al(500m buffer) 2019	-0.0943	0.0919	3.9%	0.91 [0.76, 1.09]
Lin, L., et al.	0.077	0.1595	1.5%	1.08 [0.79, 1.48]
**Subtotal (95% CI)**			**35.3%**	**0.99 [0.94, 1.03]**
Heterogeneity: Tau² = 0.00; Chi² = 4.37, df = 6 (P = 0.63); I² = 0%				
Test for overall effect: Z = 0.63 (P = 0.53)				
**6.1.4 1000m**				
Dadvand et al(1000m rhinoconjunctivitis) 2014	0.0488	0.0619	6.7%	1.05 [0.93, 1.19]
Dzhambov et al (1000m allergic rhinitis) 2021	-0.3147	0.183	1.2%	0.73 [0.51, 1.04]
Kuiper et al (1000m rhinitis) 2021	-0.0101	0.0319	11.7%	0.99 [0.93, 1.05]
Li et al(100m buffer) 2019	-0.0726	0.103	3.3%	0.93 [0.76, 1.14]
**Subtotal (95% CI)**			**22.8%**	**0.99 [0.91, 1.06]**
Heterogeneity: Tau² = 0.00; Chi² = 4.04, df = 3 (P = 0.26); I² = 26%				
Test for overall effect: Z = 0.38 (P = 0.70)				

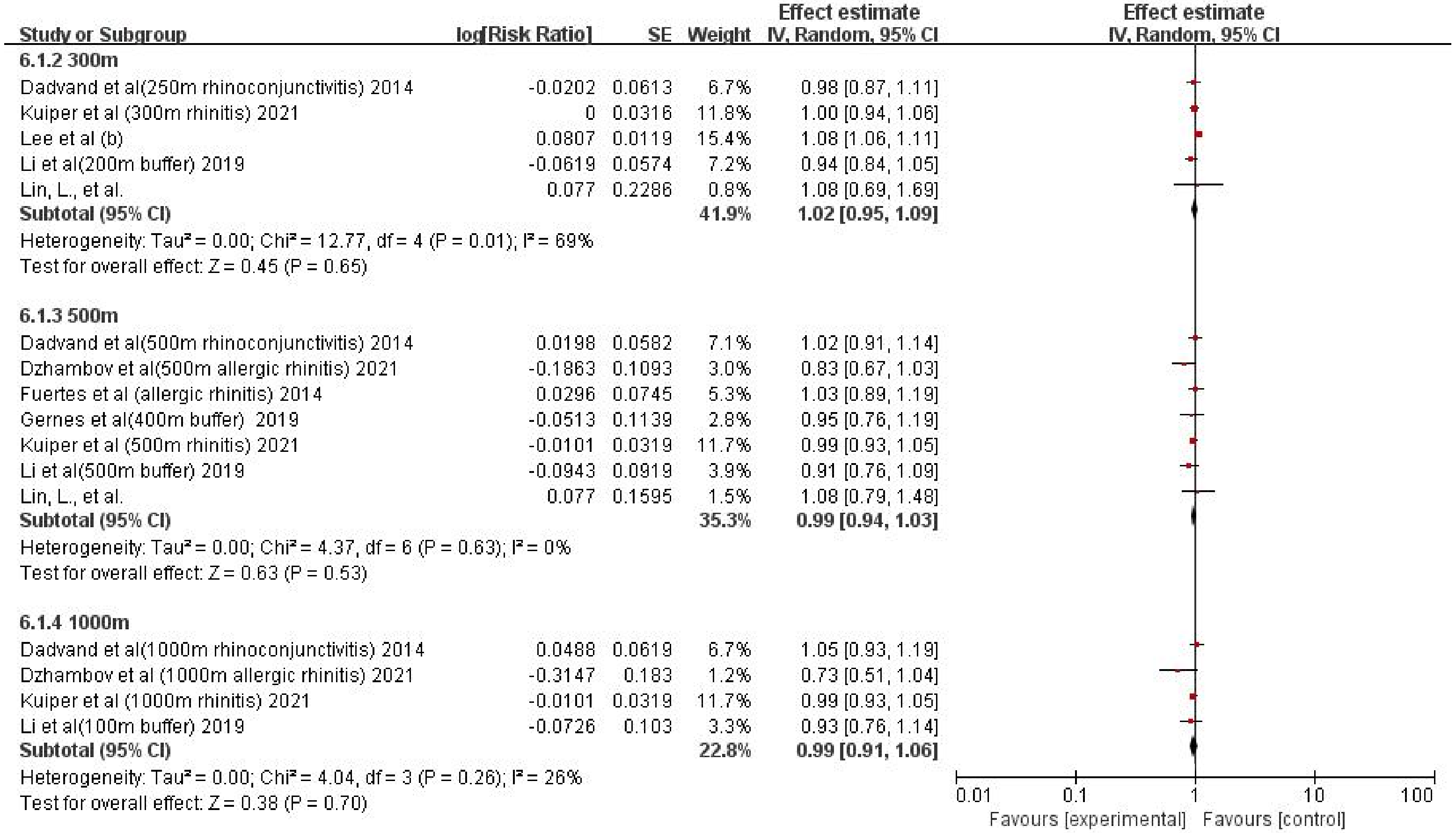

Figure S8. Buffer subgroup analysis of AR incidence/prevalence and NDVI for increments of 0.1. NDVI, Normalized Difference Vegetation Index.; AR, allergic rhinitis.

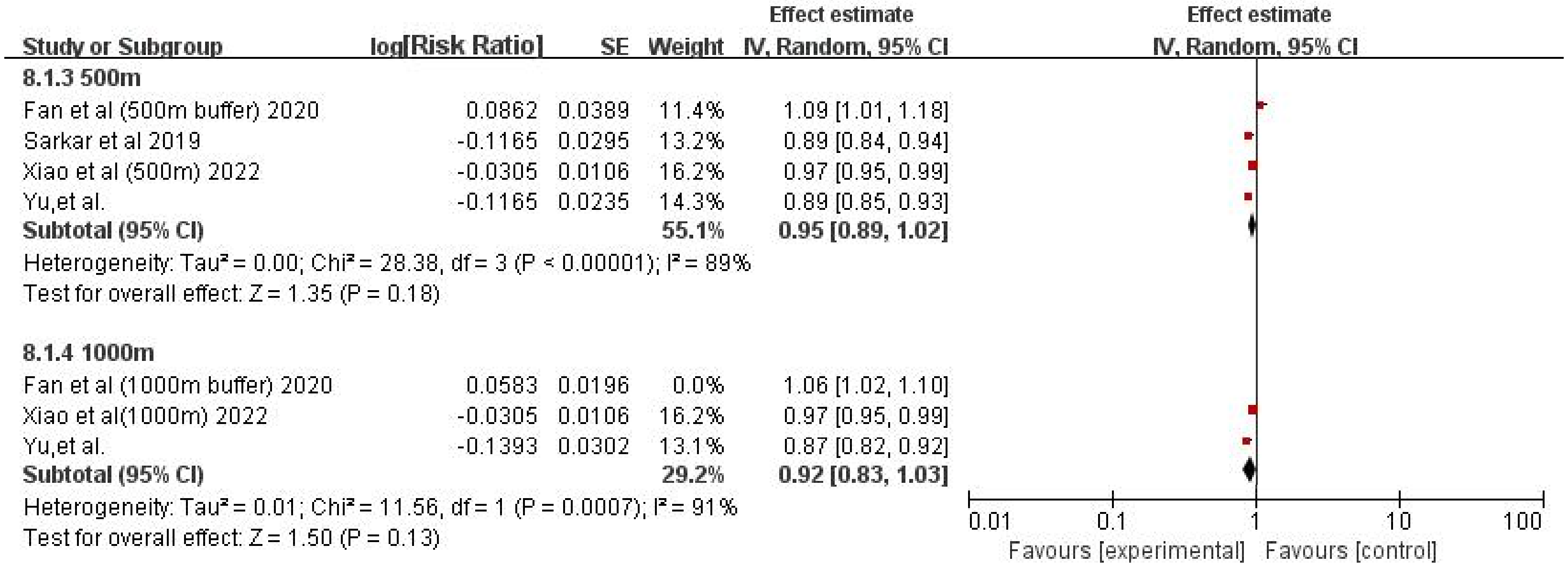

Figure S9. Buffer subgroup analysis of COPD incidence/prevalence and NDVI for increments of 0.1. NDVI, Normalized Difference Vegetation Index; COPD, Chronic Obstructive Pulmonary Diseases.

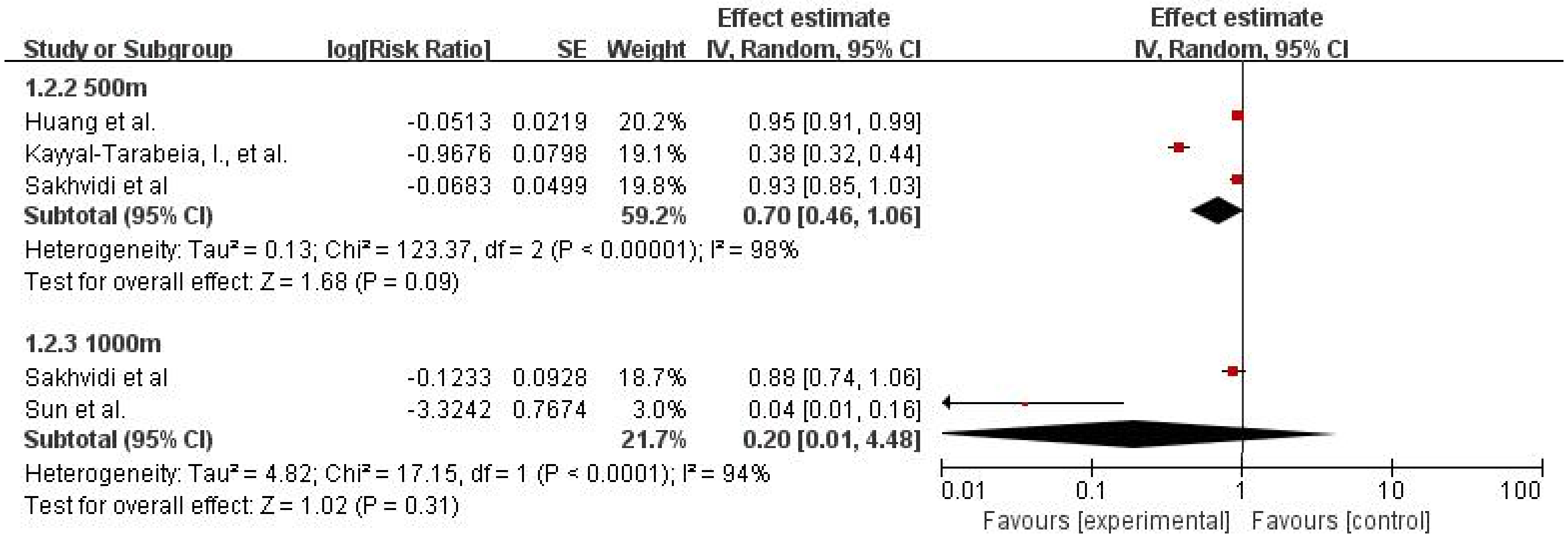

Figure S10. Buffer subgroup analysis of lung cancer incidence/prevalence and NDVI for increments of 0.1. NDVI, Normalized Difference Vegetatio n Index.

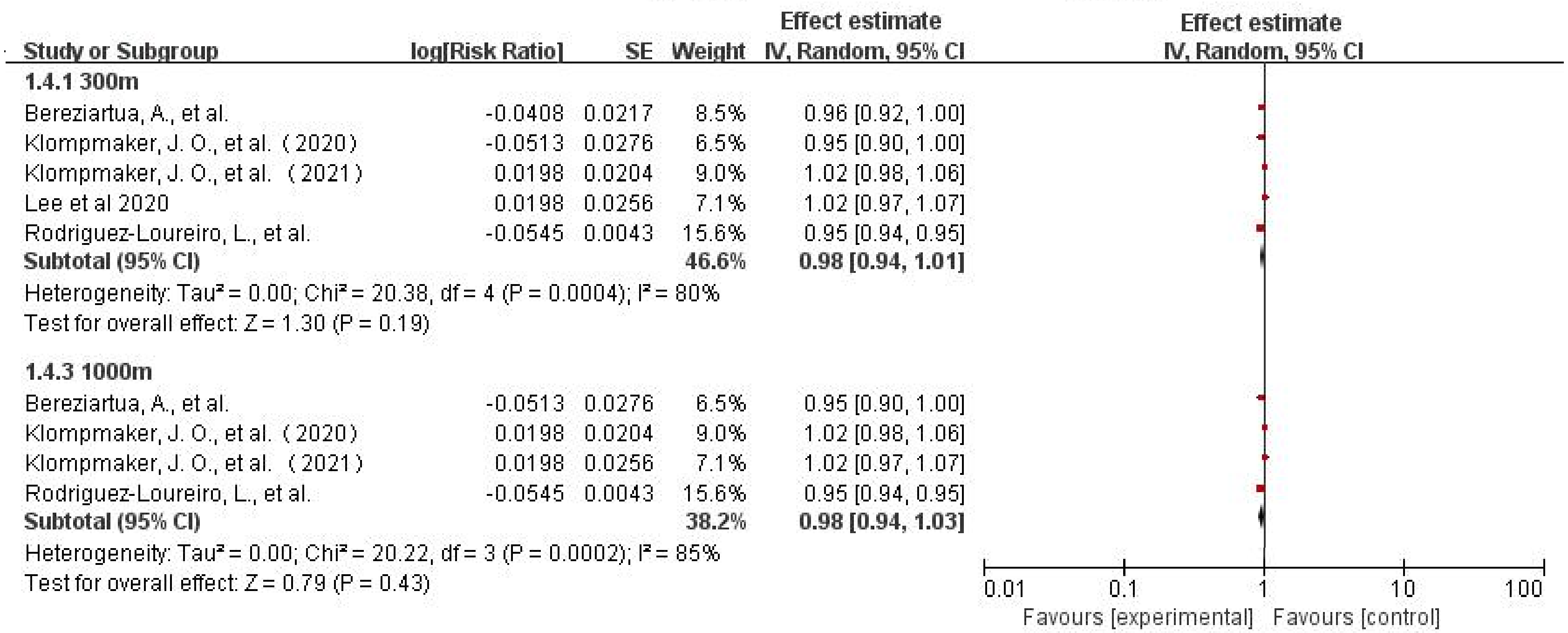

Figure S11. Buffer subgroup analysis of lung cancer mortality and NDVI for increments of 0.1. NDVI, Normalized Difference Vegetation Index.

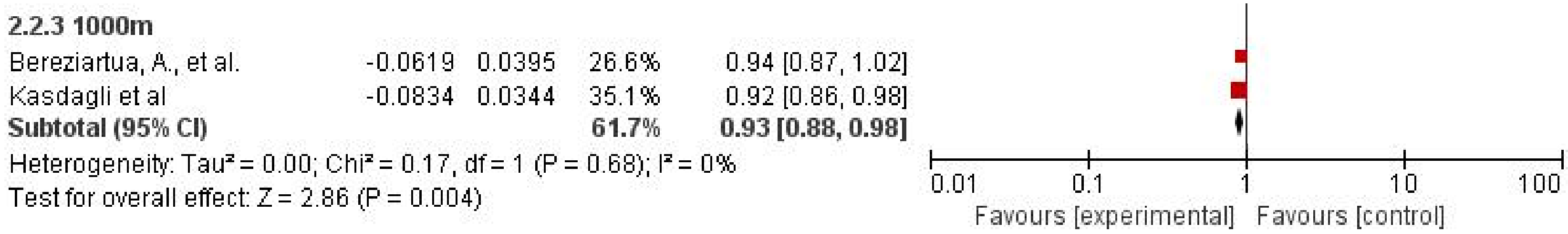

Figure S12. Buffer subgroup analysis of COPD mortality and NDVI for increments of 0.1. NDVI, Normalized Difference Vegetation Index; COPD, Chronic Obstructive Pulmonary Diseases.

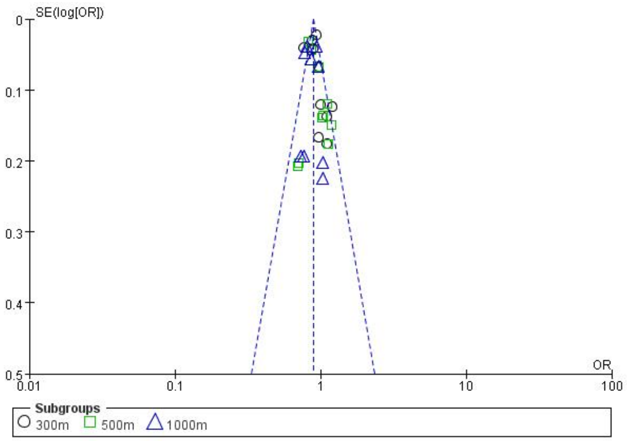

(a)

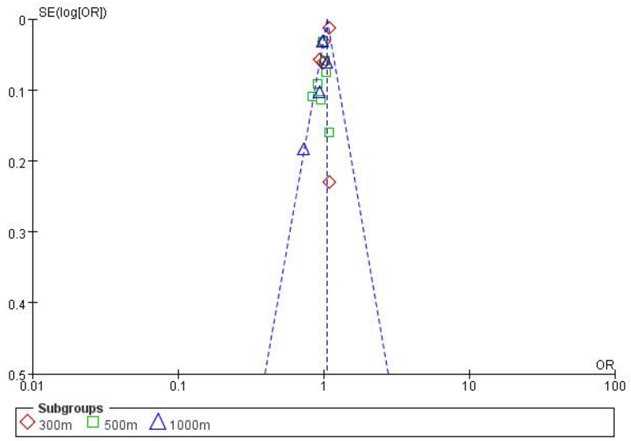

(b)

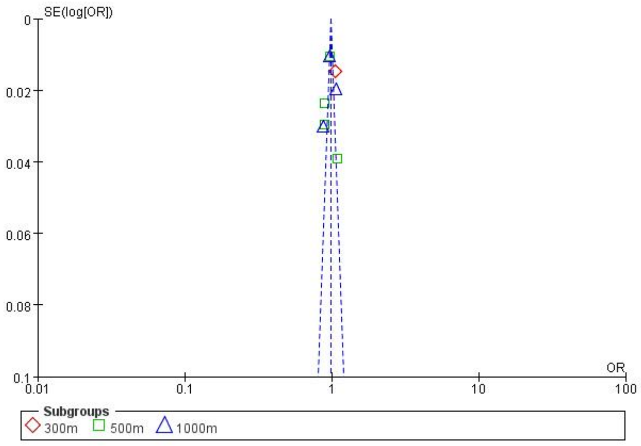

(c)

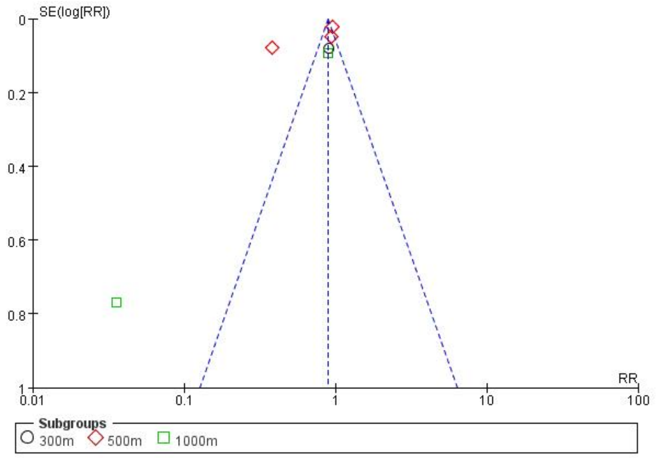

(d)

Figure S13. Funnel Plots for buffer subgroups of CRDs incidence/prevalence. (a) asthma incidence/prevalence; (b) AR incidence/prevalence; (c) COPD incidence/prevalence; (d) Lung cancer incidence/prevalence.

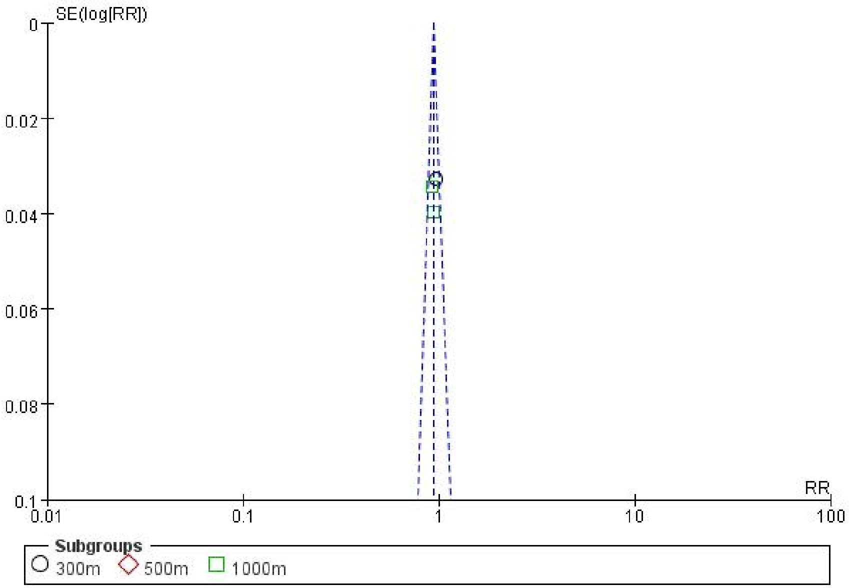

(a)

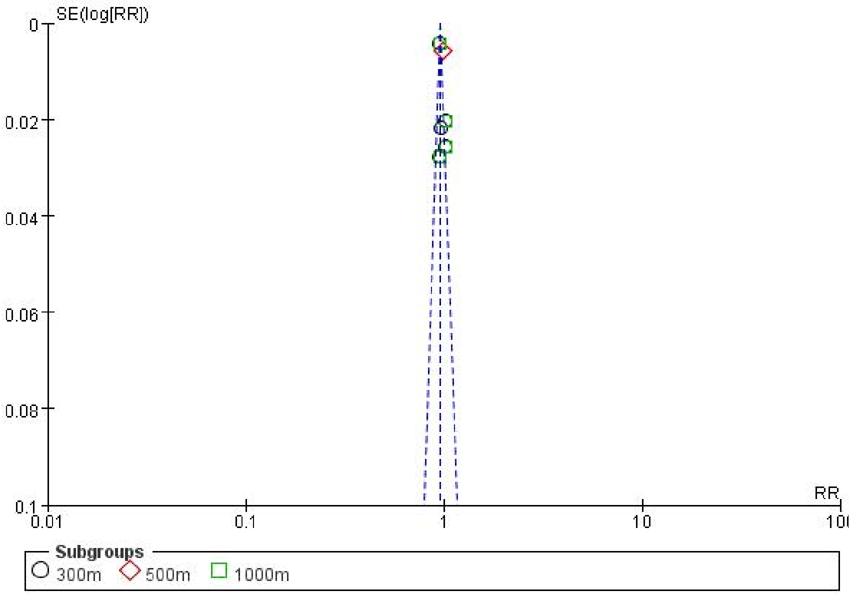

(b)

Figure S14. Funnel Plots for buffer subgroups of CRDs mortality. (a) COPD mortality; (b) Lung cancer mortality.
